# Can Small Molecules Provide Clues on Disease Progression in Cerebrospinal Fluid from Mild Cognitive Impairment and Alzheimer’s Disease Patients?

**DOI:** 10.1021/acs.est.3c10490

**Published:** 2024-02-19

**Authors:** Begoña Talavera Andújar, Arnaud Mary, Carmen Venegas, Tiejun Cheng, Leonid Zaslavsky, Evan E. Bolton, Michael T. Heneka, Emma L. Schymanski

**Affiliations:** † Luxembourg Centre for Systems Biomedicine (LCSB), University of Luxembourg, Avenue du Swing 6, L-4367 Belvaux, Luxembourg; ‡ National Center for Biotechnology Information, National Library of Medicine, National Institutes of Health, Bethesda, Maryland 20894, United States

**Keywords:** high-resolution mass spectrometry, liquid chromatography, exposomics, metabolomics, cheminformatics, bile acids

## Abstract

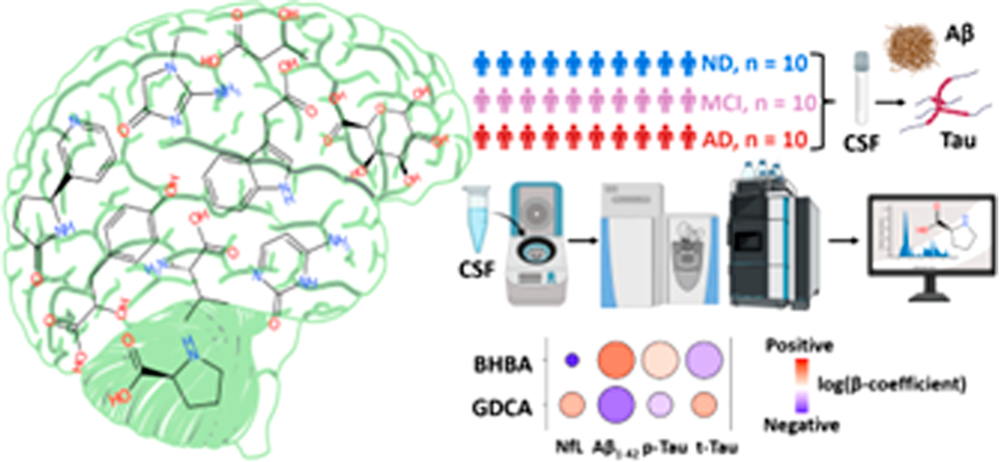

Alzheimer’s disease (AD) is a complex and multifactorial neurodegenerative disease, which is currently diagnosed via clinical symptoms and nonspecific biomarkers (such as Aβ_1–42_, t-Tau, and p-Tau) measured in cerebrospinal fluid (CSF), which alone do not provide sufficient insights into disease progression. In this pilot study, these biomarkers were complemented with small-molecule analysis using non-target high-resolution mass spectrometry coupled with liquid chromatography (LC) on the CSF of three groups: AD, mild cognitive impairment (MCI) due to AD, and a non-demented (ND) control group. An open-source cheminformatics pipeline based on MS-DIAL and patRoon was enhanced using CSF- and AD-specific suspect lists to assist in data interpretation. Chemical Similarity Enrichment Analysis revealed a significant increase of hydroxybutyrates in AD, including 3-hydroxybutanoic acid, which was found at higher levels in AD compared to MCI and ND. Furthermore, a highly sensitive target LC–MS method was used to quantify 35 bile acids (BAs) in the CSF, revealing several statistically significant differences including higher dehydrolithocholic acid levels and decreased conjugated BA levels in AD. This work provides several promising small-molecule hypotheses that could be used to help track the progression of AD in CSF samples.

## Abstract 2

The combination of non-target LC–HRMS and target LC–MS in cerebrospinal fluid reveals potential molecular markers that could indicate Alzheimer’s disease progression.

## 1 Introduction

Alzheimer’s disease (AD) is a complex and multifactorial neurodegenerative disease influenced by genetics, lifestyle, and environmental factors. AD is the most common form of dementia, and its prevalence is expected to increase from 50 million people in 2010 to 113 million by 2050 worldwide.^1,2^ AD is often divided into three stages: (1) preclinical stage characterized by normal cognitive ability, (2) prodromal stage characterized by mild cognitive impairment (MCI), and (3) dementia stage.^1,3^ Current diagnosis relies on clinical symptoms and pathological alterations indicated by biomarkers, such as reduced amyloid-β_1–42_ (Aβ_1–42_) or increased p-Tau and t-Tau concentrations in cerebrospinal fluid (CSF). Neurofilament light (NfL), a neuronal cytoplasmatic protein highly expressed in large caliber myelinated axons, has also recently emerged as a nonspecific biomarker of neurodegeneration. CSF and blood NfL levels are elevated in multiple neurodegenerative diseases, including AD, in response to axonal damage.^4,5^ AD pathology starts decades before clinical symptoms appear. Moreover, Aβ and Tau protein are quite stable in clinical AD and may not always differentiate AD from other types of dementia, leading to a high rate of misdiagnosis in the early stages.^6,7^

Since CSF is already collected for AD diagnosis, further investigation into the small-molecule signatures (e.g., via metabolomics and exposomics) could provide new insights to better understand disease progression and identify individuals at risk. CSF is the closest biological fluid to the brain such that abnormalities in this matrix are directly related to pathological changes in the brain.^8^ Despite its biological significance, the number of metabolomics/exposomics studies in CSF samples remains low. This is due to the invasive and thus precious nature of the sample (requiring lumbar puncture) combined with methodological challenges, including the lack of standard material^9^ and the relatively low chemical concentrations in CSF compared to other matrices like blood.^10^ Previous studies have revealed that alterations in various metabolomics pathways are associated with AD and MCI, including the energy metabolism, fatty acid oxidation, amino acids, and lipid biosynthesis.^11−15^ Recently, bile acids (BAs) were proposed to be involved in the AD pathogenesis^16−18^ but have not yet been explored in CSF in the context of MCI and AD.

High-resolution mass spectrometry (HRMS) coupled with liquid chromatography (LC) is a well-suited platform to study the chemical composition of CSF due to the polar nature of the matrix. The current work explores the CSF of three groups of subjects: non-demented (ND) control group, MCI due to AD (which offers the opportunity to study the disease progression), and AD. Non-target LC-HRMS was performed coupled to two different analytical columns and using various software and cheminformatics approaches for data analysis to detect small molecules potentially associated with disease progression, complemented by a highly sensitive target LC–MS method to quantify extremely low concentrations of BAs in CSF. Finally, the potential associations between clinical AD biomarkers (Aβ_1–42_, t-Tau, p-Tau, and NfL) and chemicals identified in the CSF were investigated to determine which small-molecule signatures could serve as potential biomarkers of disease progression for future investigations in a larger cohort of patients.

## 2 Materials and Methods

### 2.1 Sample Collection and Biomarker Assessment

Thirty CSF human samples (Table 1) were extracted by lumbar puncture and stored at −80 °C until analysis. Informed consent for research purposes was obtained by the ethics committee approval of the University Hospital of Bonn Ethics Commission (#279/10). Unfortunately, no lifestyle information was available. Further details are provided in the Supporting Information, Section S1.1, Figure S1, and Table S1.

**Table 1:** Clinical Characteristics of the Cohort

clinical characteristics	ND	MCI (due to AD)	AD	*p*-value^a^
	(*n* = 10)	(*n* = 10)	(*n* = 10)	
sex (female/male)	6/4	1/9	8/2	0.0055
age (years), mean ± SD	53.2 ± 16.71	66.0 ± 10.24	69.9 ± 12.96	0.0269
t-Tau (pg/mL), mean ± SD	236.9 ± 67.8	295.2 ± 68.4	549.2 ± 208.36	6.32 × 10^–5^
p-Tau (pg/mL), mean ± SD	24.5 ± 7.9	37.1 ± 10.9	95.9 ± 41.1	3.30 × 10^–6^
Aβ_1–40_ (pg/mL), mean ± SD	4043.4 ± 1467.7	5980.2 ± 1598.1	5639.7 ± 2178.5	0.0637
Aβ_1–42_ (pg/mL), mean ± SD	293.4 ± 128.8	385.8 ± 130.1	241.8 ± 109.8	0.0588
NfL (pg/mL)^b^, mean ± SD	290.2 ± 213.2	551.2 ± 240.1	1320 ± 1932.86	0.1731

a Chi-square *p*-value was computed for the categorical variable (sex). ANOVA *p*-values were calculated for the rest of characteristics.

b NfL concentrations were measured in *n* = 9 for the ND group. See Table S1 for further details.

### 2.2 Non-target and Suspect Screening

#### 2.2.1 Sample Preparation

The CSF samples were mixed with ethanol, vortexed, incubated (−20 °C), and centrifuged, as described by Song et al.^19^ The supernatant was evaporated to dryness and reconstituted using Milli-Q water:MeOH:MeCN (2:1:1, v/v/v). Ten internal standards (ISs) were added (Table S2), and pooled quality control (QC) samples were prepared according to recent recommendations (Supporting Information, Section 1.2, and Figure S2). The ISs (spiked in all CSF samples) were employed to check the instrument performance but were not included in the subsequent data analysis. The sample preparation method was first tested on artificial CSF samples (HelloBio Ltd., UK) using the same protocol as above, with the addition of 10 μL of a mixture containing 121 polar chemical standards (50 μM) to serve as reference standards later. Further details are given in the Supporting Information (S1.3, Table S3 and Figure S3).

#### 2.2.2 Instrumental Analysis

Analytical measurements were performed on an Accela LC system coupled to a Q Exactive HF mass spectrometer (both Thermo Scientific) using electrospray ionization in both positive (+) and negative (−) modes. BEH C18 reversed phase (RPLC) (1.7 μm, 2.1 × 150 mm) and SeQuant ZIC-pHILIC polymer (HILIC) (5 μm, 150 × 2.1 mm) columns were used, in separate runs, to detect a broader range of chemicals. The HRMS was operated in a full-scan profile mode with the scan range 60–900 *m*/*z* using the methods described in Talavera-Andújar et al.^20^ The QC samples were analyzed prior to the first sample and every three or four sample injections.

#### 2.2.3 Disease-Specific Chemical Lists

**Figure 1:**
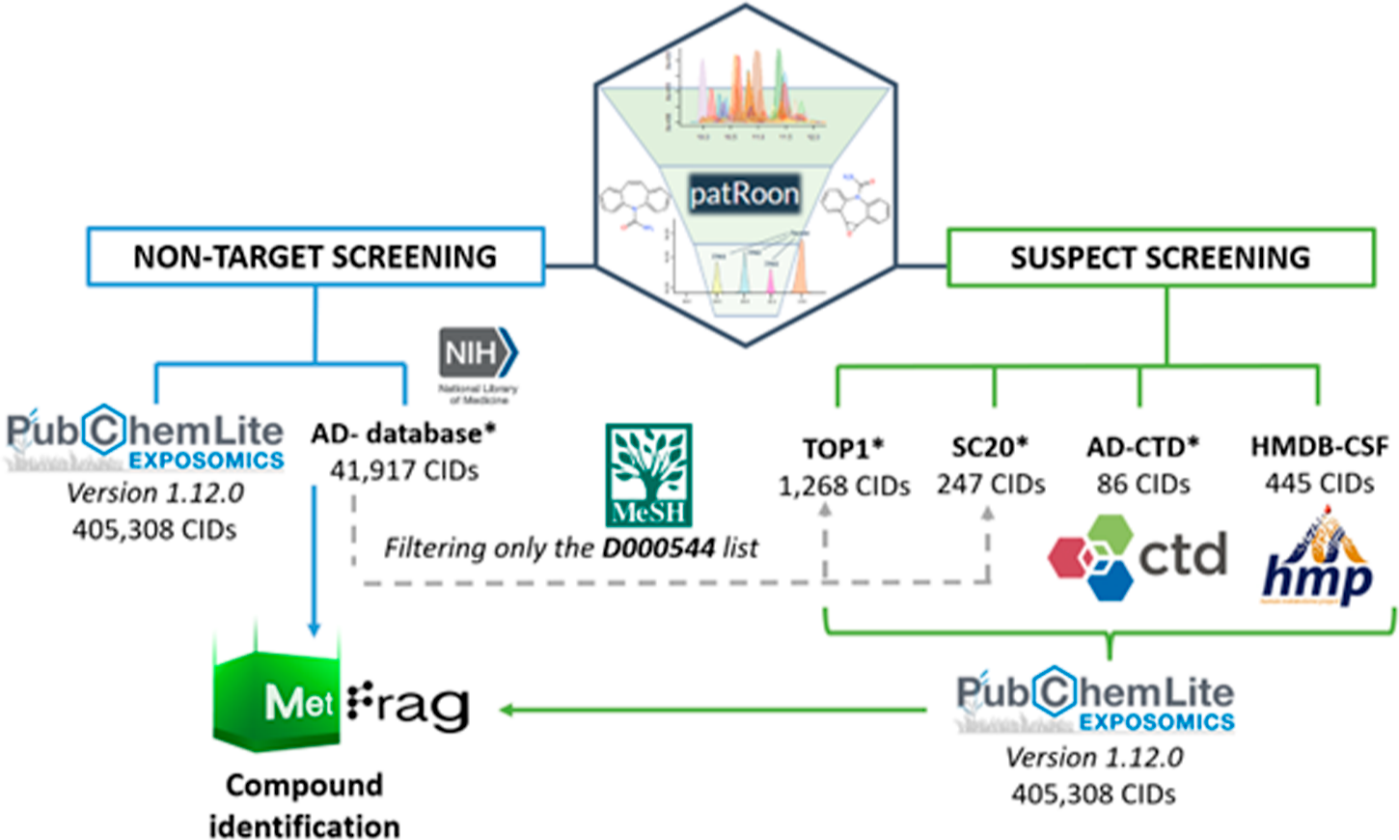
Databases and suspect lists employed for the non-target screening (left) and the suspect screening (right) analysis with patRoon. *Databases/suspect lists created for the purpose of this study.^27−29^ See the main text, S1.4, and Table S4 for more details. CID: PubChem Compound IDentifier.

New disease-specific database (*AD-database*) and suspect lists (*TOP1*, *SC20*, and *AD-CTD*) were created to explore the CSF metabolome and exposome of MCI and AD subjects (Figure 1). First, the *AD-database* was created through literature mining,^21^ integrating chemicals co-occurring with 27 Medical Subject Headings (MeSH) related to AD or symptoms, given in Table S4. This database was filtered to create smaller lists based on reverse neighboring relations (*TOP1*) and co-occurrence scores (*SC20*), as detailed in S1.4 and Figures S4 and S5. A list of chemicals (*AD-CTD*) specifically related to AD in the Comparative Toxicogenomic Database (CTD) was also extracted from PubChem.^22^ These lists were complemented with the publicly available CSF Human Metabolome database (*HMDB-CSF*)^23,24^ and *PubChemLite* for Exposomics (*PCL*).^25,26^ The associated code and lists are available on GitLab^27^ and Zenodo,^28^ respectively.

#### 2.2.4 Data Processing

Raw LC-HRMS files were converted to .mzML using ProteoWizard MSConvert (version 3.0.20331.3768aa6e9 64-bit) and analyzed with MS-DIAL (version 4.90),^30^ MS-FINDER (version 3.52),^31,32^ and patRoon (version 2.1.0)^33,34^ (see S1.5 for details). MS-DIAL was used to perform non-target analysis via MSP-formatted libraries (MSMS-Public-Pos-VS17 and MSMS-Public-Neg-VS17 for (+) and (−) modes, respectively) using the parameters in Table S5. Features without a tentative candidate via MS-DIAL were uploaded to MS-FINDER to annotate them via in silico fragmentation (Figure S6). patRoon was employed for both suspect and non-target screening (Figure 1); all scripts including parameters and settings are available in GitLab.^27^

After the analysis with MS-DIAL and patRoon, peak intensity tables were used to filter features based on the QC samples (see S1.5). The remaining features were manually checked and annotated using three different sets of criteria tailored to the three different data analysis approaches. Briefly, MS-DIAL features were annotated based on the library spectral match using the *Dot product* (0–100). Level 2a was assigned with *Dot product* ≥ 70 and ≥3 ion fragments matching with a known structure in the library, while Level 2b was assigned to features with the same requirements but unknown structure in the library (these are spectra that are commonly detected in samples belonging to unknown structures). Level 3a was assigned to features with 50 ≤ *Dot product ≤ 70* and ≥3 ion fragments matching, while Level 3b or Level 3c was assigned when <3 ion fragments were matching with known and unknown structures, respectively. Level 3d and Level 3e corresponded to features annotated via MS-FINDER, which is detailed in Table S6.

For features identified through patRoon non-target screening, the individualMoNAscore (0-1) was employed for the annotation. Level 2a was assigned when individualMoNAscore > 0.9. Level 3a was considered when the score was in the range of 0.7–0.9 and Level 3b when 0.4–0.7, as previously described.^20,35^ Chemicals identified by patRoon suspect screening were automatically annotated following predefined rules specified in the handbook.^36^

Identifications were considered Level 1 when the match between the standard and tentative candidate (in the CSF) yielded a *SpectrumSimilarity* score ≥ 0.7 and the retention time (RT) shift was <1 min. *OrgMassSpecR*(37−39) was used to calculate spectral similarity. Xcalibur Qual Browser (version 4.1.31.9) was used to check the RT and extract the MS/MS information.

Peak intensity tables of the annotated features were preprocessed with MetaboAnalyst 5.0^40,41^ by filtering (interquartile range option), normalization by sum, log transformation (base 10), and Pareto scaling. Finally, Level 1–3 compounds were classified using the HMDB disposition ontology,^42,43^ PubChem pathways information^44^ in the PubChem classification browser, and/or literature associated with PubChem records via co-occurrence scores.^21^ See S1.5 and GitLab^27^ for details.

### 2.3 Target Screening of BAs

The target study of BAs used an Agilent 1290 LC system (Waters C18 column, 1.7 μm, 2.1 × 150 mm) coupled with a Sciex 7500 QQQ MS in a multiple-reaction monitoring (MRM) mode with (−) detection, as described by Han et al.^45^ A 10 μM standard mixture (94 BAs in total, Table S7) was prepared in an IS solution of UDCA-D_4_ in MeCN. Next, 60 μL of each CSF sample was mixed with 140 μL of the IS solution, vortexed, sonicated, centrifuged, then dried, and dissolved in 40 μL of 50% MeCN. Fifteen microliters per sample was injected in the LC–MS system. BA concentrations were calculated by interpolating the constructed IS calibrated linear–regression curves of individual BAs, with the peak area ratios measured from injections of the sample solutions.

### 2.4 Statistical Analysis

One-way analysis of variance (ANOVA) with post-hoc Tukey’s honestly significant difference (HSD) test for multiple comparisons was computed via R (*aov* and *TukeyHSD* functions). Compounds with post-hoc test *p*-values <0.05 were considered statistically significant. Chemical Similarity Enrichment Analysis (ChemRICH)^46,47^ was performed to explore differentially regulated clusters of metabolites between ND-AD, MCI-AD, and ND-MCI. Linear multiple regression analysis (via *lm* function in R) was used to analyze the relationship between the biomarker concentrations (Aβ_1–40_, Aβ_1–42_, p-Tau, t-Tau, and NfL) and the relevant compounds found in CSF. Plots were created with R, Excel, and GraphPad Prism (version 10.1.0).

## 3 Results and Discussion

### 3.1 Non-target Characterization of CSF in MCI and AD

#### 3.1.1 Compound Annotation and Classification

**Figure 2:**
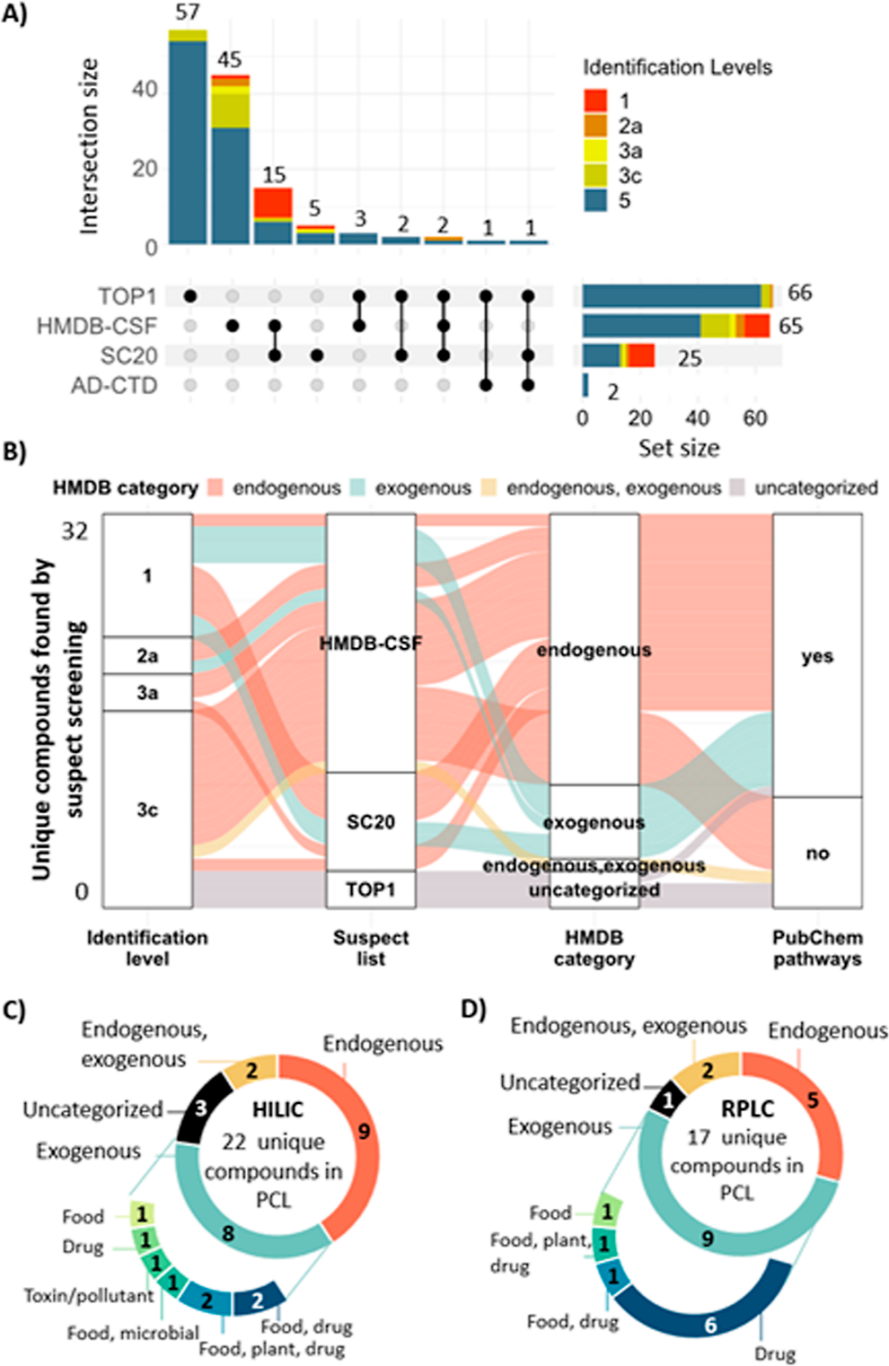
(A) UpSet plot representing the number of annotated features in each suspect lists plus overlap across lists using HILIC. See Figure S8 for RPLC results. (B) Alluvial plot showing the HMDB categories of the features annotated by each suspect screening approach. RPLC and HILIC annotations were combined, and duplicates were removed prior to plotting (32 unique compounds in total). The presence (or not) of PubChem pathways information is indicated in the last column. (C,D) Pie charts showing the classification of the compounds identified by patRoon non-target screening with PCL by HILIC (C) and RPLC (D). See Table S8.1 for detailed information about Level 1–3 compounds and Table S8.2 for Level 5 compounds.

**Figure 3:**
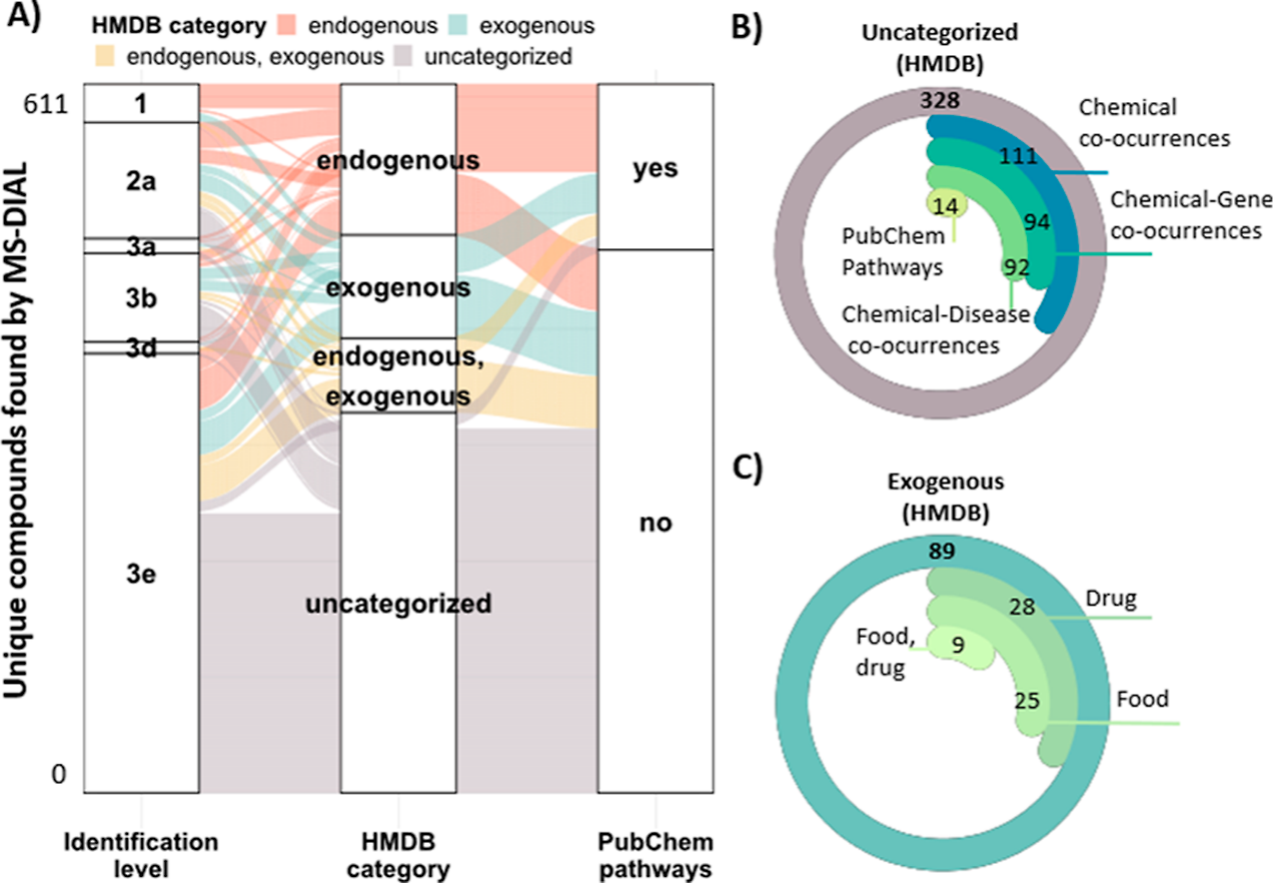
(A) Alluvial plot showing the HMDB categories of the Level 1–3 features annotated using MS-DIAL MSPs (+) and (−) libraries. The presence (or not) of PubChem pathways information is indicated in the last column. This plot represents the 611 unique identifications found by RPLC and HILIC. (B) Pie chart representing how many of the uncategorized compounds by HMDB (gray bar) have literature knowledge via PubChem classification browser. (C) Pie chart showing the exogenous subcategories (by HMDB) of the unique MS-DIAL identifications found using RPLC and HILIC.

The CSF samples were analyzed by RPLC and HILIC and annotated with patRoon (suspect and non-target screening) and MS-DIAL. The total number of Level 1–3 annotations can be found in the Supporting Information: Table S8 for patRoon suspect screening, Table S9 for patRoon non-target screening, and Table S10 for MS-DIAL. Figure 2 summarizes the patRoon annotations (the equivalent UpSet plot for RPLC is given in Figure S8), while Figure 3 shows the MS-DIAL annotations.

Overall, the overlap between the different suspect lists was low (Figure 2A), confirming the need for the complementary suspect screening approaches applied here. While most of the unique features were found in the largest lists (*TOP1*, *HMDB-CSF*), the highest confidence features (Level 1) were exclusively found in *HMDB-CSF* (metabolites previously identified in CSF) and *SC20* (chemicals associated with AD through literature mining). Thus, the filtering method utilizing co-occurrence scores to generate the *SC20* list was more effective than the reverse neighboring relations approach for generating the *TOP1* list. In stark contrast to the previous work with PD,^20^ the *AD-CTD* list did not reveal any confident annotations but only two in total. Interestingly, a considerably higher number of unique features were observed in the *HMDB-CSF* suspect list using HILIC (45 features, Figure 2A), compared to RPLC (15 features, Figure S8), suggesting that HILIC is a more effective chromatographic approach for CSF analysis likely due to the matrix’s polarity.

The origin of the annotated chemicals is explored in Figure 2B, revealing that most of the annotated chemicals are endogenous. Since exogenous species are typically present at trace levels compared to endogenous metabolites, it is challenging to capture both concurrently; furthermore, detection in CSF requires exogenous species to cross the blood–brain barrier (BBB), which regulates the passage of substances to the CNS and CSF.^48^Figure 2B also shows why it can be challenging to distinguish between endogenous and exogenous compounds when interpreting exposomics results, as this is often difficult to disambiguate in various resources. Some exogenous compounds according to the HMDB are associated with PubChem Pathways information, suggesting a potential endogenous nature. Examples include amino acids such as histidine, tryptophan, and phenylalanine which can be synthesized endogenously by humans or obtained exogenously from the diet. Interestingly, only compounds annotated at the lowest confidence level shown (Level 3c) from the *TOP1* list could not be verified with information available in either HMDB or PubChem Pathway.

Annotation with *PubChemLite* revealed 22 (HILIC, Figure 2C) and 17 (RPLC, Figure 2D) unique features between Levels 1 and 3 (Table S9). The same compounds were identified using the *AD-database* except for metoprolol acid (Level 2a) and l-beta-homolysine (Level 3a). Despite only 18,677 chemicals overlapping between *PubChemLite* and the *AD-database* (Figure S9), most of the features annotated in the CSF samples were within this overlap. Although both databases (*PubChemLite* and the *AD-database*) focus on the exposome and include primarily exogenous compounds (Figure S10), most of the annotations by the HILIC method were categorized as endogenous (Figure 2C), and the annotation results were not biased by the nature of the database. The RPLC method captured a smaller number of endogenous chemicals (Figure 2D). Drugs constituted the primary subcategory among the exogenous compounds identified by RPLC, whereas HILIC revealed a more diverse array of exogenous substances (Figure 2C).

Using MS-DIAL and the public MSPs, 611 unique compounds were annotated between Levels 1–3 (excluding Levels 2b and 3c, as their structure is unknown in the libraries), including 271 (RPLC) and 340 (HILIC) (Table S10). Overall, HILIC was the preferred LC approach for the CSF analysis with better chromatographic separation on average for compounds detected in both modes. In general, Level 1–2a compounds tended to be endogenous, while Level 3e was mainly uncategorized with no PubChem pathways information, as noted above (Figure 3A). Interestingly, some of the uncategorized compounds in the HMDB had PubChem information (Figure 3B). Drugs and food (Figure 3C) constituted the main exogenous subcategories of the MS-DIAL annotations.

#### 3.1.2 Statistically Significant Chemicals in MCI and AD

Cheminformatics and statistical approaches were used to identify significant chemicals potentially associated with disease progression. Twelve Level 1–2a features were identified as statistically significant (Tukey’s HSD post-hoc *p*-value < 0.05), summarized in Table 2 and Figure S11A–L. Full results, including Level 3 features, are available in Tables S8–S10.

**Table 2:** Statistically Relevant Compounds Found Using MS-DIAL and patRoon^a^

								post-hoc *p*-values			ANOVA
chemical name	rt (min)	*m*/z**	LC mode	IL	HMDB category	PubChem pathways	library/database/suspect list	MCI-AD	ND-AD	ND-MCI	*p*-value
valine	6.99	118.0862	HILIC (+)	1	exogenous	yes	PCL/AD-database	0.9991	0.0320*	0.0293*	0.0156*
proline	6.93	116.0706	HILIC (+)	1	endogenous	yes	PCL/AD-database	0.1432	0.7350	0.0303*	0.0325*
*N*-acetylhistidine	8.14	198.0872	HILIC (+)	2a	endogenous, exogenous	no	MSDIAL-MSPs	0.4226	0.3859	0.0374*	0.0477*
3-hydroxybutanoic acid (BHBA)	5.92	103.0396	HILIC (−)	1	endogenous	yes	MSDIAL-MSPs	0.0042*	0.0150*	0.8637	0.0030*
indole-3-acetic acid (IAA)	13.66	176.0706	RPLC (+)	1	endogenous	yes	PCL/AD-database/SC20/HMDB-CSF	0.7248	0.0390*	0.0064*	0.0061*
4-hydroxyphenyl lactic acid (4-HPLA)	6.46	181.0495	HILIC (−)	2a	endogenous, exogenous	no	MSDIAL-MSPs	0.9935	0.0574	0.0455*	0.0285*
adenine	3.42	136.0617	HILIC (+)	1	endogenous	yes	MSDIAL-MSPs	0.9958	0.0213*	0.0174*	0.0091*
cytosine	5.90	112.0505	HILIC (+)	2a	endogenous	yes	MSDIAL-MSPs	0.0216*	0.6122	0.1575	0.0253*
galacturonic acid	12.24	193.0342	HILIC (−)	1	endogenous	yes	MSDIAL-MSPs	0.9534	0.0753	0.0403*	0.0305*
threonic acid	10.48	135.0291	HILIC (−)	1	endogenous	no	MSDIAL-MSPs	0.4707	0.3345	0.0361*	0.0457*
cotinine	1.76	177.1021	HILIC (+)	1	endogenous	no	MSDIAL-MSPs	0.0915	0.8744	0.0320*	0.0284*
diazepam	17.11	285.0787	RPLC (+)	2a	exogenous	no	MSDIAL-MSPs	0.0373*	0.6052	0.2429	0.0447*

a Only Level 1 and Level 2a annotations are included. *p-value < 0.05. **Adducts were [M + H]^+^ for (+) and [M – H]^−^ for (−) mode. IL: identification level. See Tables S8–S10 and Figure S11 for detailed information.

Significantly altered levels of valine and proline were observed, which is consistent with prior research indicating disrupted amino acids pathways in AD,^11,49,50^ potentially due to the alteration of different neurotransmitters. Valine (Figure S11A) was significantly decreased in MCI and AD groups compared to the ND group, in line with previous studies reporting decreased valine levels in AD, associated with impaired neurotransmission and cognitive function.^11,13^ Furthermore, adenine (Figure S11G) was also significantly reduced in both MCI and AD, compared to ND. These results are consistent with previous studies performed in mice,^51^ indicating that the purine metabolism pathway may be altered in AD and potentially plays an important role in the pathogenesis.

3-Hydroxybutanoic acid (BHBA, Figure S11D) was found with statistically higher levels in the AD group compared to the other two groups. BHBA is the most abundant ketone in the human circulation and may be involved in many brain functions including neurotransmission, neuroinflammation, and myelination.^52^ The higher levels in the AD group may be due to increased fat degradation and thus ketone formation as a physiological response to energy shortage in the brain.^52−54^ Ketogenic diets, which increase BHBA concentrations, may also contribute to increased levels, but dietary information was not available for these samples.

Statistically higher levels of indole-3-acetic acid (IAA, Figure S11E) were found in the MCI and AD groups compared to the ND group, aligning with a recent study reporting significantly higher levels of IAA in the plasma of AD patients compared to control subjects.^55^ IAA was previously found upregulated in the CSF of MCI.^56^ IAA has shown proinflammatory and prooxidant effects,^57^ potentially contributing to neurodegeneration, such that higher levels in MCI might serve as an inflammatory indicator, as previously suggested.^55^

Galacturonic acid (Figure S11I) was found with statistically higher levels in the MCI group compared to the ND group, along with a similar but non-significant trend in the AD group compared to the ND group. Galacturonic acid is the major component of pectin, which is found in fruits and vegetables, where the higher levels found in both AD and MCI groups could be due to increased BBB permeability, associated with the dementia status. Hence, this could be a biomarker of BBB dysfunction.^58^

Significantly higher levels of cotinine, the main metabolite of nicotine, were found in the MCI group compared to the ND group (Figure S11K). Interestingly, tobacco smoking has been correlated with a lower incidence of AD. Cotinine has shown to prevent memory loss and inhibit Aβ aggregation without the toxicity and addictive properties of its precursor (nicotine).^59,60^ However, since no lifestyle information was available in this study, this cannot be interpreted further at this stage.

#### 3.1.3 Statistically Relevant Chemical Clusters

**Figure 4:**
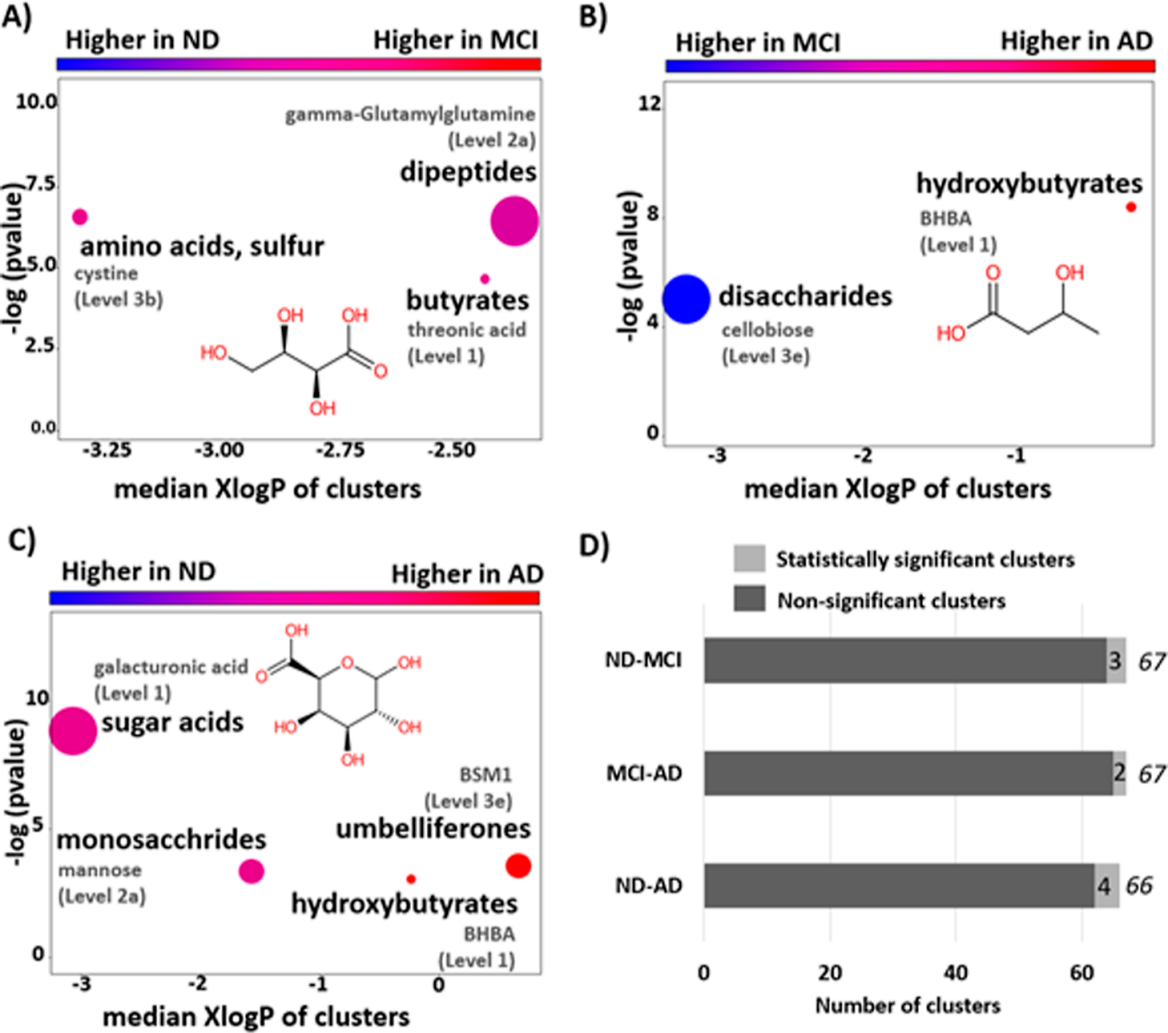
ChemRICH analysis between (A) ND-MCI, (B) MC-AD, and (C) ND-AD. Enrichment *p*-values are given by the Kolmogorov–Smirnov test. Each dot represents a significantly altered cluster of chemicals (*p*-value <0.05). Dot size is proportional to the number of metabolites in the cluster. The node color scale shows the proportion of increased (red) or decreased (blue) metabolite levels in MCI (A) or AD (B,C). Purple-color nodes have both increased and decreased metabolites. Names of key metabolites in each cluster are displayed in gray; structures are shown only for Level 1 key metabolites. (D) Bar plot representing the total number of clusters identified in each of the comparisons. BSM1 = 2-(2-methyl-4-oxochromen-5-yl)acetic acid. See Table S11 for further details. Chemical structures were drawn with CDK Depict.^61^

ChemRICH analysis was performed to facilitate the biological interpretation of the non-target results, as it accounts for both endogenous and exogenous chemicals. Unlike other common approaches such as pathway enrichment analysis, ChemRICH’s *p*-values do not rely on the size of a background database (e.g., KEGG),^46^ avoiding overrepresentation issues. Instead, ChemRICH is study-specific with a self-contained size. The analysis here considered Level 1–3 annotations; the results are given in Figure 4 (including key annotations in each cluster) and Table S11.

Different significant chemical clusters were found across the three comparisons. The ND-MCI comparison (Figure 4A) revealed significant alterations in three chemical clusters: sulfur amino acids, dipeptides, and butyrates, with threonic acid (Figure S11J) the key metabolite of the latter. The MCI-AD comparison (Figure 4B) revealed two significant clusters: disaccharides (decreased in AD) and hydroxybutyrates (increased in AD, with key metabolite BHBA, discussed above). This last cluster was also statistically altered by comparing ND-AD (Figure 4C). Umbelliferones (increased in AD), monosaccharides, and sugar acids were also significant in ND-AD. While the total number of chemical clusters identified was almost the same in the three cases (Figure 4D), only one significant cluster (hydroxybutyrates) overlapped between MCI-AD and ND-AD.

### 3.2 Target Study of BAs in CSF of MCI and AD

**Figure 5:**
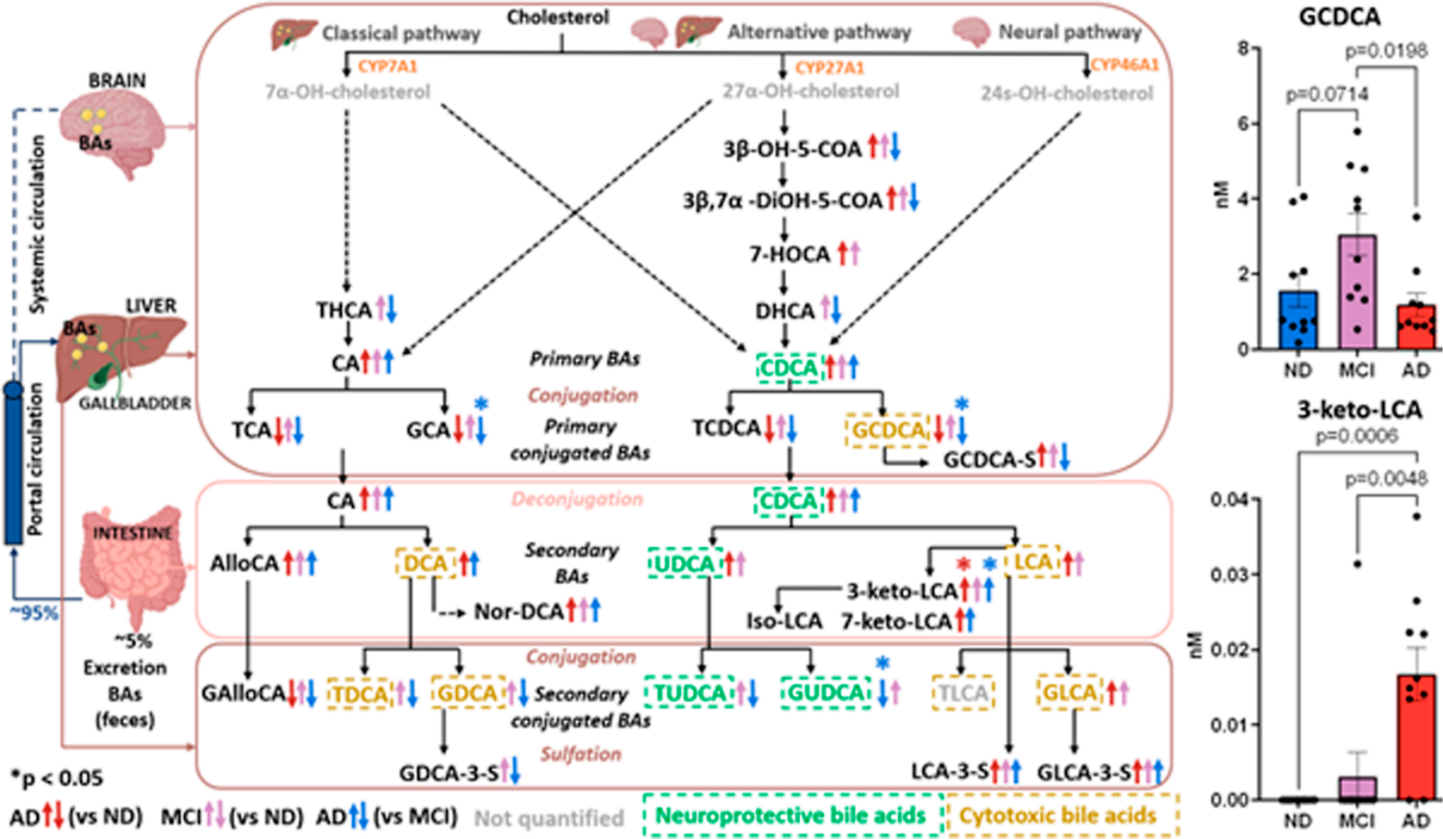
Schematic representation of BAs (and precursors) quantified in this study. Primary BAs are synthesized from cholesterol through different pathways. The classical pathway in the liver (top left) is responsible for the most BA synthesis. The alternative pathway (top middle) occurs in other tissues besides the liver, such as the brain. The neural pathway (top right) takes place in the brain and is responsible for the majority of cholesterol turnover in the CNS. Primary BAs, after conjugation with taurine or glycine in the liver, are secreted into the bile and transported to the gut where the gut bacteria deconjugate the conjugated BAs, generating secondary BAs (middle box). Most of the BAs (95%) are reabsorbed in the ileum via portal circulation to the liver. Only a small portion escapes the enterohepatic circulation and reaches the systemic circulation. Arrows indicate the higher (↑) or lower (↓) concentrations. No arrow means no differences between groups. Right box plots show the mean concentration with standard error of the mean of GCDCA (top) and 3-keto-LCA (bottom). *p* = Tukey’s HSD post-hoc *p*-value. Note that *p* < 0.1 is displayed although only *p* < 0.05 is considered statistically significant in this work, marked with an asterisk (*) in the scheme. Abbreviations: 3-keto-LCA, dehydrolithocholic acid; 3β,7α-DiOH-5-COA, 3β-7α-DiOH-5 cholestenoic acid; 3β-OH-5-COA, 3β-OH-5-cholestenoic acid; 7-HOCA, 7α-hydroxy-3-oxo-4-cholestenoic acid; 7-keto-LCA, 7 ketolithocholic acid; AlloCA, allocholic acid; CA, cholic acid; CDCA, chenodeoxycholic acid; DCA, deoxycholic acid; DHCA, 3α,7α-dihydroxycholestanoic acid; GAlloCA, glycoallocholic acid; GCA, glycocholic acid; GCDCA, glycochenodeoxycholic acid; GDCA, glycochenodeoxycholic acid; GLCA, glycolithocholic acid; GUDCA; glycoursodeoxycholic acid; HCA, hyocholic acid; LCA, lithocholic acid; Nor-DCA, nordeoxycholic acid; TCA, taurocholic acid; TCDCA, taurochenodeoxycholic acid; TDCA, tauoursodeoxycholic acid; THCA, 3α,7α,12α-trihydroxycholestanoic acid; TUDCA, tauroursodeoxycholic acid; UDCA, ursodeoxycholic acid. Adapted from refs (16, 64, 65–67).

Of the 94 BAs included in the targeted method, 35 were quantified in the CSF samples (Table S12). An overview of these results is given in Figure 5, and significant results are marked with an asterisk. The identification of BAs in CSF implies a possible source through either systemic circulation uptake or local synthesis within the brain.^62^ While a previous study quantified BA precursors in the CSF of AD patients,^63^ to our knowledge, this is the first time that BAs are quantified in CSF in the context of MCI and AD.

Primary BAs are synthesized from cholesterol through different pathways (top of Figure 5). While the classical pathway in the liver is responsible for most BA synthesis, the brain uses the alternative and neural pathways to clear cholesterol, leading to the production of BAs.^18,62^ The intermediates of the alternative pathway are explained in S2.2 and Figure S12. The primary BAs, cholic acid (CA), and chenodeoxycholic acid (CDCA) presented a non-significant higher trend in the MCI and AD groups compared to the ND group (Figure S13). In contrast, the glycine conjugated primary BA glycocholic acid (GCA) and glycochenodeoxycholic acid (GCDCA), top right of Figure 5, showed significantly lower concentrations in the AD group compared to the MCI group. Elevated concentrations of these two BAs were previously reported in AD plasma samples compared with control subjects.^17,68^ Therefore, CSF concentrations may not always reflect circulating BA levels. Furthermore, the conjugates with taurine, taurocholic acid (TCA), and taurochenodeoxycholic acid (TCDA) exhibited the same low trend in the AD group compared to the others, without statistical significance (Figure S13).

The secondary and cytotoxic BAs, lithocholic acid (LCA), and deoxycholic acid (DCA) increased non-significantly in MCI and AD subjects compared with ND subjects (Figure S14). Higher levels of LCA and DCA were previously noted in AD blood samples,^17,64,68^ suggesting LCA as a putative biomarker for AD.^68^ Interestingly, 3-keto-LCA, the major metabolite of LCA, was found with statistically higher concentrations in AD compared to MCI and ND (bottom right of Figure 5). This is a microbial metabolite, not previously reported in CSF, which could reflect the importance of the microbiota–gut–brain axis (MGBA) in neurodegeneration.

As observed with the primary conjugated BAs, the secondary conjugated BAs showed lower trends in the AD group compared to the MCI group (Figure S15). Significantly lower concentrations of GUDCA were found in the AD group compared to the MCI group, which is in line with previous studies identifying GUDCA as a potential blood marker for early diagnosis that could predict the onset of AD or MCI with 2–3 years and 90% of accuracy.^69,70^ This highlights the importance of the MCI group in studying early disease biomarkers. While the average GCDCA concentration across the 30 samples was 1.92 nM, the average of GCDCA-S was 871.56 nM. This trend was also observed for GDCA (0.60) and GDCA-S (866.73); see Figure S16. Sulfation, catalyzed by SULT2A1 in humans, is an important detoxification pathway of BAs. The resulting sulfated BAs are less toxic and more soluble, leading to reduced intestinal absorption and enhanced fecal and urinary excretion.^71,72^ This suggests that the brain may utilize sulfation to mitigate the BA toxicity, as SULT2A1 is expressed not only in the liver but also in the brain.^71^

To explore whether the observed dysregulation of conjugated BAs in AD is linked to enzymatic differences in taurine and glycine conjugation, the ratios GDCA:DCA, TDCA:DCA, GCA:CA and TCA:CA were calculated (Figure S17) as previously described.^64^ A significant decrease in GDCA:DCA was found in AD compared to MCI, suggesting a change in the processes involving glycine conjugation in the liver. This is in contrast with a previous study in blood where no significant changes were observed.^64^

The presence of secondary cytotoxic BAs with higher concentrations in the MCI and AD groups supports a previously published hypothesis^64^ that gut microbiome dysregulation leads to increased production of cytotoxic secondary BAs and derivatives such as 3-keto-LCA. Moreover, elevated hydrophobic BAs in blood, such as DCA and CDCA, can alter the BBB permeability,^17,64^ which might explain some of these results.

### 3.3 Correlation between Altered Metabolites in CSF and Classical Biomarkers

**Figure 6:**
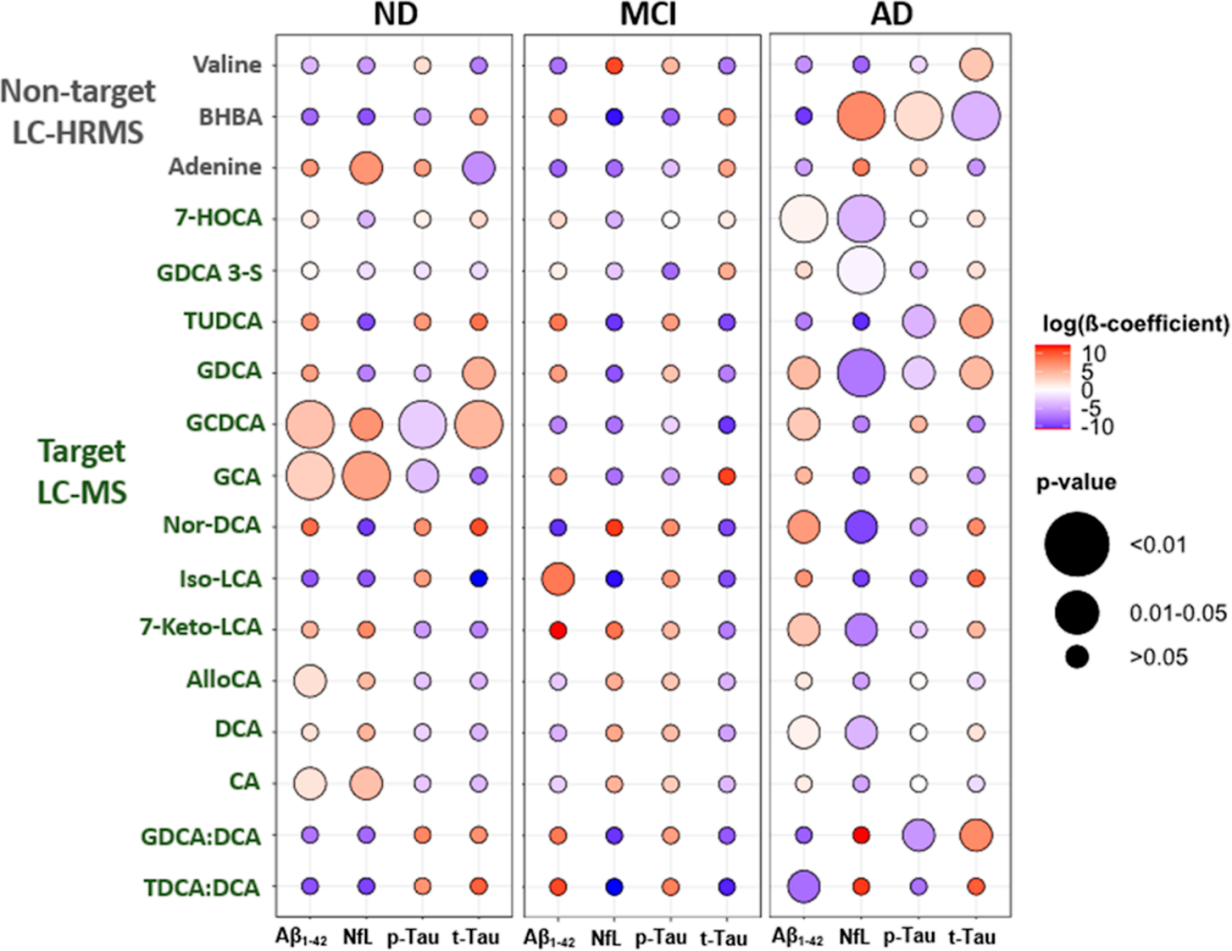
Associations between the statistically relevant compounds found in CSF, by non-target (first three rows) and target screening, and Aβ_1–42_, NfL, t-Tau, and p-Tau concentrations. Color represents the log-transformed β-coefficients. Positive and negative associations are indicated by the red and blue colors, respectively. Note that only compounds with a statistically significant association are illustrated. See Tables S13–S15 for further details. BHBA: 3-hydroxybutanoic acid; see Figure 5 for the rest of abbreviations.

Finally, the correlation between the altered metabolites and the concentrations of diagnostic biomarkers Aβ_1–42_, p-Tau, t-Tau, and NfL in CSF was explored (Figure 6 and Tables S13–15). Age, sex, and Aβ_1–40_ concentrations were considered as covariates to compute the different linear models. Although a positive β-coefficient indicates a positive association between two variables (e.g., t-Tau and valine levels), an association does not necessarily imply causation.

Since previous studies have shown that metabolic changes in AD blood and CSF were associated with the disease status and pathological alterations (e.g., brain atrophy),^11,73−75^ correlating the identified metabolites in CSF with the classical AD biomarkers might reveal additional insights.^74^ The results here show multiple significant associations between the altered compounds found in CSF from AD (e.g., BHBA, GDCA, and Nor-DCA) and Aβ, Tau, and NfL levels (right panel of Figure 6). In contrast, the associations in the MCI group were weaker (middle panel of Figure 6), with only one significant association (Iso-LCA and Aβ_1–42_). The ND group (left panel of Figure 6) presented various significant associations with the classical biomarkers, most strikingly for GCDCA and GCA. Some of them correlate (positively or negatively) as in the AD group (e.g., GDCA, GCDCA, and Aβ_1–42_ were positively correlated in both groups) potentially due to a disease-independent relationship, as explained by Jacobs et al.^75^

Briefly, significant positive associations were found for BHBA with NfL and p-Tau in the AD group with a significant negative association for t-Tau. The ND group exhibited the opposite but non-significant correlations. This disparity in associations may suggest disease-specific patterns in AD. Multiple significant associations were found between the quantified BAs and the classical biomarkers in AD. In short, the neurotoxic GCDCA, GDCA, and the ratio GDCA:DCA were significantly and positively associated with t-Tau in the AD group, the first association in line with a previous work performed in serum from AD.^73^ Additionally, a statistically significant negative association was observed between the TDCA:DCA ratio and Aβ_1–42_ in the AD group, which might be associated with higher cerebral amyloid burden.^11^

## 4 Future Perspectives

The identification of chemicals with statistically higher levels in the MCI compared to the ND (e.g., galacturonic acid, IAA, 4-HPLA) shows the importance of this group for the early identification of individuals at risk. However, external factors, including diet, medication, and exercise, may account for some of the observed chemical differences across groups, such that more information about these factors would enhance the interpretation of findings. Notably, one AD patient exhibited high outlier levels for NfL and BHBA compared with the other patients in the group (Figure S18), and it would be interesting to investigate whether this is due to AD pathology or to environmental factors such as a ketogenic diet, as previously discussed.

The HILIC LC method (Table 2) appears to be the most suitable method for future non-target experiments. Since most of the enriched clusters (Figure 4) are highly hydrophilic (log*P* < −1), an expansion of the non-target methods to explore the hydrophobic part (lipidome) of the CSF could reveal extra information in future efforts. Overall, while MS-DIAL provided a higher number of annotated chemicals, the combination of different software and suspect lists enhanced the annotation of a variety of chemicals, increasing the general understanding of the CSF metabolome/exposome. Furthermore, the identification of some chemicals in this study (e.g., galacturonic acid, threonic acid, *N*-acetylhistidine, and some of the BAs) could help expand the current HMDB-CSF database, as they are not yet included in this resource.

This study highlights the possible role of the microbiota−gut−brain−axis (MGBA) in the disease progression, as some metabolites found altered in MCI and/or AD, such as 3-keto-LCA, are produced by the human microbiome. However, the role of BAs in CSF needs to be further investigated as the link between peripheral and central BAs is poorly understood. Matching samples of CSF, plasma, and feces would be needed to study the influence of the microbiota composition.

Although the low sample size can be considered a limitation of this study, the development of novel non-target cheminformatics approaches together with the highly sensitive target study of BAs in CSF provides valuable insights into this complex matrix (CSF) and disease progression. Multiple significant molecules were found in both MCI and AD compared to ND. Moreover, some significant associations between the altered metabolites and the CSF biomarkers in AD were observed. Further studies in a larger cohort of samples will be necessary to validate the promising hypothesis and results presented here to determine which of these small molecules may reveal insights into disease progression.

## Data Availability

The code functions and files associated with this manuscript are provided in the ECI GitLab repository (https://gitlab.lcsb.uni.lu/eci/AD-CSF). The PubChemLite database (https://doi.org/10.5281/zenodo.6936117) and database/suspect lists created here (https://doi.org/10.5281/zenodo.8014420) are available for download on Zenodo.^28,29^

## Supporting Information Available

Additional details regarding materials and methods (S1), results and discussion (S2) plus Figures S1−18: scatter dot plots of biomarkers; the four types of QC samples; overview of the aCSF samples; screenshot of acetylcholine chemical-disease co-occurrences; scatter plot of the co-occurrence score; MS-DIAL annotation decision tree; HMDB curation; UpSet plot for RPLC features; Venn plot AD-database vs PCL; bar plot showing the total number of CIDs in AD-database and PCL; scatter dot plots for significant non-target chemicals; bar plots of BA precursors; bar plots of the primary conjugated and unconjugated BAs; bar plots of the secondary unconjugated BAs; bar plots of the secondary conjugated BAs; bar plots of the sulfated BAs; bar plots of various bile acid ratios; and scatter plots of NfL and BHBA (PDF)
Tables S1−S15: Detailed clinical information of the studied groups; internal standards; chemical standards; summary of MeSH codes; MS-DIAL parameters; identification confidence level system; bile acid standards; annotated compounds by suspect screening; annotated compounds by patRoon non-target screening; annotated compounds by MS-DIAL; ChemRICH results; bile acids quantified in the CSF samples; linear models with only ND; linear models with only MCI; and linear models with only AD information (XLSX)

## Notes

Ethics Declarations: Informed consent for use of samples and data for research purposes was given with the local ethics committee approval (University Hospital of Bonn Ethics Commission #279/10). This work does not contain identifiable data of the subjects or any other specific individual person’s data.
