# Supplementary material for: Can Small Molecules Provide Clues on Disease Progression in Cerebrospinal Fluid from Mild Cognitive Impairment and Alzheimer’s Disease Patients?
Source: Environ Sci Technol. 2024 Feb 19;58(9):4181–92. doi: 10.1021/acs.est.3c10490 (PMC10919072; doi:10.1021/acs.est.3c10490)
Supplement: Supplementary file 1 — es3c10490_si_001.pdf [file es3c10490_si_001.pdf]

Supporting Information

Can Small Molecules Provide Clues on Disease Progression in Cerebrospinal Fluid from Mild Cognitive Impairment and Alzheimer's Disease Patients?

Begoña Talavera Andújar[1*], Arnaud Mary[1], Carmen Venegas[1], Tiejun Cheng[2], Leonid Zaslavsky[2], Evan E. Bolton[2], Michael T. Heneka[1], Emma L. Schymanski[1*]

1. Luxembourg Centre for Systems Biomedicine (LCSB), University of Luxembourg, Avenue du Swing 6, L-4367 Belvaux, Luxembourg

2. National Center for Biotechnology Information, National Library of Medicine, National Institutes of Health, Bethesda, MD 20894, USA

***Corresponding authors:** begona.talavera@uni.lu and emma.schymanski@uni.lu

ORCIDs: BTA: 0000-0002-3430-9255, AM: 0000-0002-6597-766X, CV: 0000-0002-8902-4986, TC: 0000-0002-4486-3356, LZ: 0000-0001-5873-4873, EEB: 0000-0002-5959-6190, MTH: 0000-0003-4996-1630, ELS: 0000-0001-6868-8145

This supporting information file contains:

- 27 pages and 18 figures - see detailed contents on page S2;
- Details of additional supporting information files (15 tables in a separate excel file, plus links to code and repositories) – see page S3.

Table of contents

Section 1: Material and methods S4
Section 1.1: Human CSF biomarker analysis S4
  Figure S1 .... S5
Section 1.2: CSF sample preparation S6
  Figure S2 .... S7
Section 1.3: Validation of the sample preparation method with artificial CSF (aCSF) S8
  Figure S3 .... S9
Section 1.4: Development of a specific database and suspect lists for AD S9
  Figure S4 .... S11
  Figure S5 .... S11
Section 1.5: Data processing S12
  Figure S6. .... S13
  Figure S7 .... S14
Section 2: Results and discussion S15
Section 2.1: Non-target characterization of CSF in MCI and AD S15
  Figure S8 .... S15
  Figure S9. .... S15
  Figure S10 .... S16
  Figure S11 .... S17
Section 2.2: Target study of BAs in CSF of MCI and AD S18
  Figure S12 .... S18
  Figure S13 .... S19
  Figure S14 .... S20
  Figure S15 .... S21
  Figure S16 .... S22
  Figure S17 .... S23
Section 3: Future Perspectives S24
  Figure S18 .... S24

Supporting Tables

Tables S1-S15 are available in the excel file (**Supporting_tables.xlsx**).

Supporting Figures

Figures S1-S18 are provided in this document.

Other Supporting Information

The code for this study is available on GitLab: (https://gitlab.lcsb.uni.lu/eci/AD-CSF)

Additional tables are available on Zenodo: (https://doi.org/10.5281/zenodo.8014420)

Section 1: Material and methods

Section 1.1: Human CSF biomarker analysis

On the day of the analysis, samples were thawed at room temperature and the tubes were vortexed for 5–10 seconds. t-Tau was quantified without dilution in a single run, while $A\beta_{1\text{-}42}$, $A\beta_{1\text{-}40}$, and p181-Tau were quantified in a separated run after manually diluting the CSF three times with Sample Diluent 1 (Fujirebio #292617). NfL was quantified separately after manually diluting the CSF four times. CSF samples were poured into Hitachi sample cups (Fujirebio #80351) and placed in the Lumipulse G600II analyzer (Fujirebio #703380). Biomarkers levels were measured automatically by the analyzer, via the chemiluminescent enzyme immunoassay technology, using the corresponding Lumipulse G immunoreaction cartridges (Fujirebio #230312, #230350, #230336, #231524, and #81426), after controlling the cartridges by using the manufacturer's corresponding calibrators (Fujirebio #230329, #230367, #230343, #231531, and #81413) and controls (Fujirebio #230237, #230220, #231548, #231548, and #81414), following the manufacturer's instructions. **Figure S1** shows the distributions of the biomarker levels across the three groups under study. See **Table 1** (main manuscript) and **Table S1** (**Supporting_tables.xlsx**) for detailed clinical information about the CSF samples.

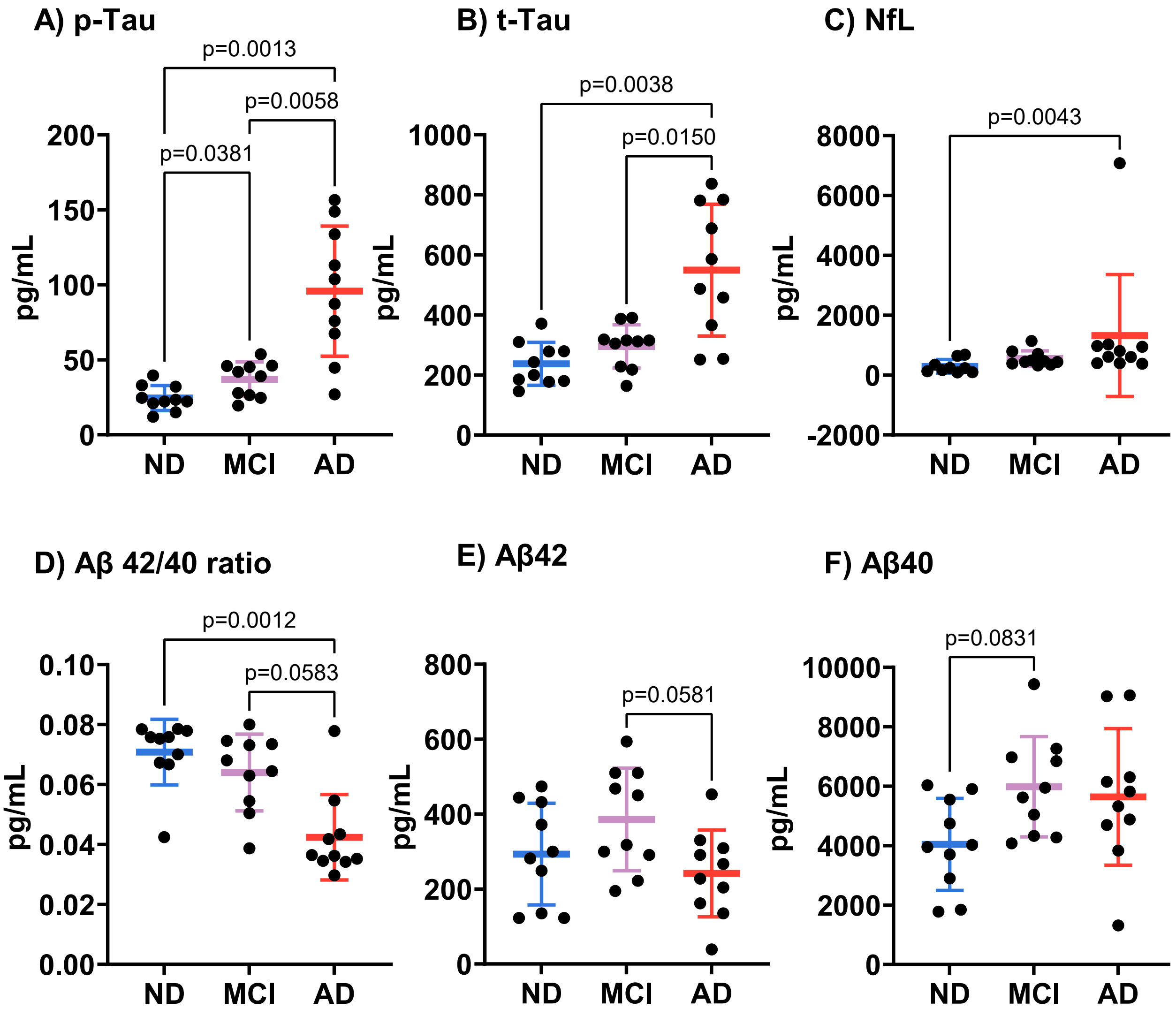

Figure S1: *Scatter dot plots showing mean concentration ± standard deviation (SD) of (A) p-Tau, (B) t-Tau, (C) NfL, (D) ratio A$\beta_{42/40}$, (E) Aβ_{42} and (F) Aβ_{40} across the three groups investigated. p= Dunnett's T3 multiple comparisons p-value. Note that p-values <0.1 are displayed although only p-values <0.05 are considered statistically significant in this work.*

Section 1.2: CSF sample preparation

The sample preparation was adapted from Song *et al*[1]. First, samples were thawed at room temperature. Then, 100 µL of CSF was mixed with 400 µL of ethanol (EtOH) containing the internal standard (IS) mixture (**Table S2**). Next, samples were vortexed (30-60 seconds), incubated at -20°C for one hour, and centrifuged at 13,000 rpm for 15 minutes at 4°C. The clear supernatant was placed into new Eppendorf tubes and transferred to the Labconco CentriVap to evaporate the solvents until dryness (-4 °C for 24h-48h). Afterwards, samples were reconstituted with 100 µL of Milli-Q water:MeOH:MeCN (2:1:1, v/v/v), vortexed (30-60 seconds), sonicated (15 minutes) and centrifuged (same conditions as previously). Finally, the clear supernatant was transferred to LC-MS vials. To test the system suitability, blank extraction samples were prepared using 100 µL of Milli-Q water instead of CSF.

Additionally, four different pooled Quality Control (QC) samples were prepared following published guidelines/recommendations[2,3]. First, a system suitability QC sample (QC-SS) was prepared by mixing 5 µL of each sample, to condition the system. For the data analysis, three separate QC samples were prepared by mixing 15 µL of each sample within a group: (1) QC-AD (prepared only with AD samples), (2) QC-MCI (prepared only with MCI samples) and (3) QC-ND (prepared only with ND samples). **Figure S2** represents the QC samples prepared during this study schematically.

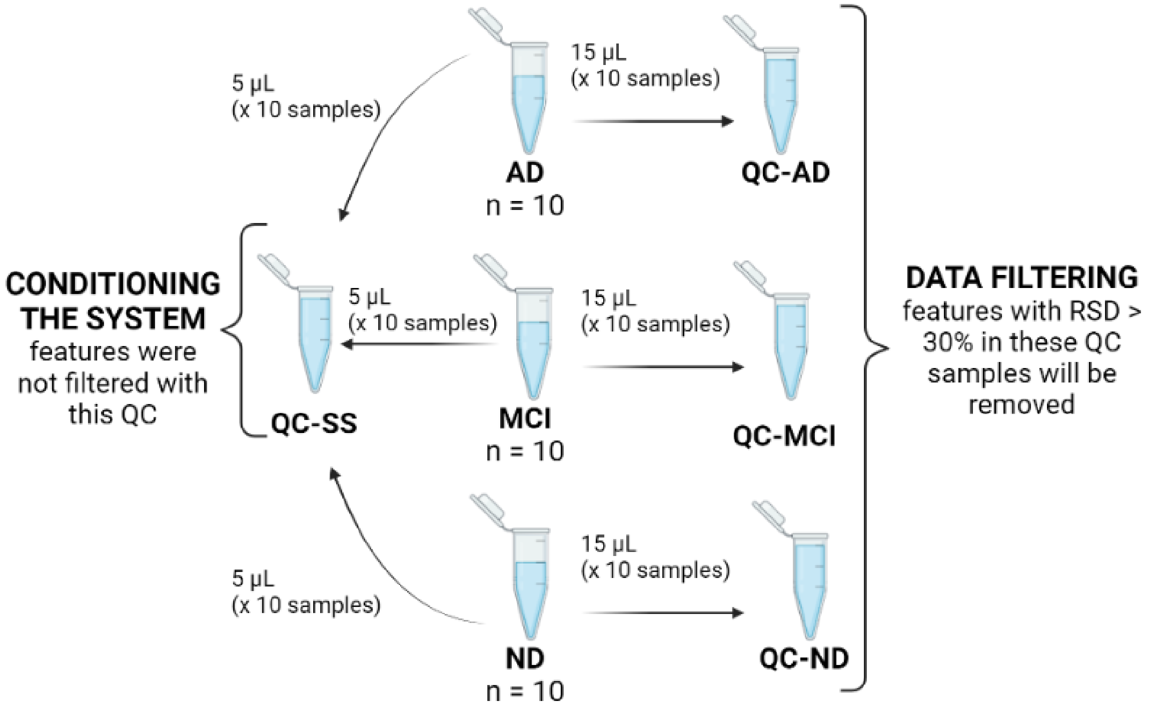

Figure S2: *Representation of the four types of QC samples prepared during the study: (left) QC-SS, (right) QC-AD, QC-MCI and QC-ND. QC-SS was prepared for conditioning the system while the other three QC samples were used to filter the number of features identified.*

Section 1.3: Validation of the sample preparation method with artificial CSF (aCSF)

To test the suitability of the extraction method four different types of samples were prepared:

- CSF_IS: aCSF aliquots (100 µL) spiked with the IS mix in EtOH (0.5 mg/L). These chemicals were employed to check the instrument performance. See **Table S2** for detailed information.
- CSF_free: aCSF aliquots (100 µL) without IS.
- CSF_STD: aCSF aliquots (90 µL) spiked with 10 µL of a chemical standard (STD) mix (50 µM). These compounds will serve as reference STDs to check the level 1 compounds in the actual CSF samples. Compounds were spiked in aCSF and not in any other solvent to mimic the compounds in the actual samples. See **Table S3** for detailed information about the STD.
- CSF_STD_IS: aCSF aliquots (90 µL) spiked with the IS and the STD mix, same concentrations as described for the previous samples.

The four types of samples were prepared in triplicate and extracted following the procedure described in **S1.2**. Briefly, 100 µL of aCSF were used for CSF_IS and CSF_free samples, and 90 µL for CSF_STD, CSF_STD_IS and Extraction_blank samples. Then, 10 µL of the STD mix (50uM) was added only in CSF_STD, CSF_STD_IS and Extraction_blank samples. Next, 400 µL of EtOH were added to the CSF_STD, CSF_free and Extraction_blank samples and 400 µL EtOH containing the IS were added only in the CSF_IS and CSF_IS_STD samples. Samples were vortexed (30 seconds), incubated (-20°C for 1 hour), centrifuged (13000 rpm for 15 minutes at 4°C) and evaporated to dryness. Finally, samples were reconstituted with 100 µL of Milli-Q water:MeOH:MeCN (2:1:1, v/v/v), vortexed, centrifuged and the supernatant was transferred into LC-MS vials.

All the compounds were manually checked to ensure that the method was adequate, allowing the identification of most of the compounds in aCSF. **Figure S3** shows the samples that were prepared schematically.

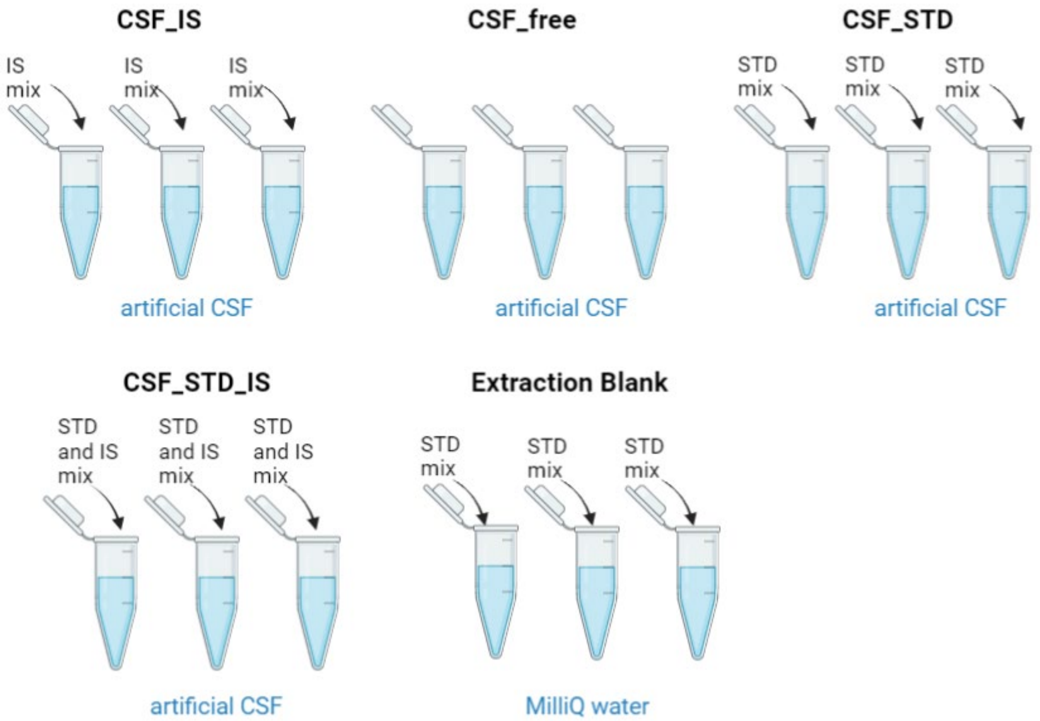

Figure S3: *Overview of the samples prepared to check the extraction method with aCSF. IS: internal standard. STD: reference standard. Note that the chemical information for "IS mix" and "STD mix" is provided in* ***Table S2*** *and* ***Table S3****, respectively.*

Section 1.4: Development of a specific database and suspect lists for AD

PubChem functionality relating Medical Subject Headings (MeSH) information to chemicals was used to create a database focused on AD and other CNS diseases and health problems related to AD. All CIDs co-occurring with the 27 selected MeSH terms (**Table S4**) were merged and mapped to the parent CIDs (to obtain the neutral, desalted forms needed for mass spectral screening) via the PubChem Identifier Exchange Service[4]. Duplicates were removed and the *webchem*[5] package was used to add the chemical information necessary for the analysis in patRoon (exact mass, formula, chemical name, SMILES, InChI and InChIKey). The resulting list, "*AD-database*", contained 41,917 different CIDs and was used in the patRoon non-target screening approach (Non_target_pos_AD_database.R and Non_target_neg_AD_database.R - see GitLab repository[6]).

Moreover, to further explore the chemicals related to AD (MeSH code D000544), two different filtering approaches based on Zaslavsky *et al.*[7] were tested to obtain smaller lists of chemicals

that could be used as suspect lists in patRoon. The "*TOP1*" suspect list of 1,268 CIDs was created by selecting chemicals with D000544 as first neighbor based on the reverse neighboring relations. Acetylcholine (CID = 187) is one chemical present in this list (**Figure S4**). The "*SC20*" list was created by truncating chemicals with less than 1/20th of the maximum Co-Occurrence Score, which decreases quickly with the number of CIDs, as shown **in Figure S5**. The remaining 321 CIDs were mapped to parents as described above to yield a final "SC20" suspect list contains 247 CIDs.

As described in the manuscript, a list of 86 CIDs ("*AD-CTD*") specifically related to AD in the Comparative Toxicogenomic Database (CTD), was extracted via CTD integration within PubChem and used as a suspect list. In addition, *PubChemLite* (405,308 CIDs)[7] was employed as a database, along with the CSF Human Metabolome Database ("*HMDB-CSF*")[8,9]. This list of 445 small molecules is integrated into the Human Metabolome Database (HMDB) and was downloaded as SDF file, then converted to csv format to be used as a suspect list (*HMDB-CSF*) in patRoon (Suspect_pos_neg.R, see GitLab[6]).

All code used to create these lists is freely available in the GitLab repository[6], while the chemical lists created *in house* are available in the Zenodo repository[10] for further use.

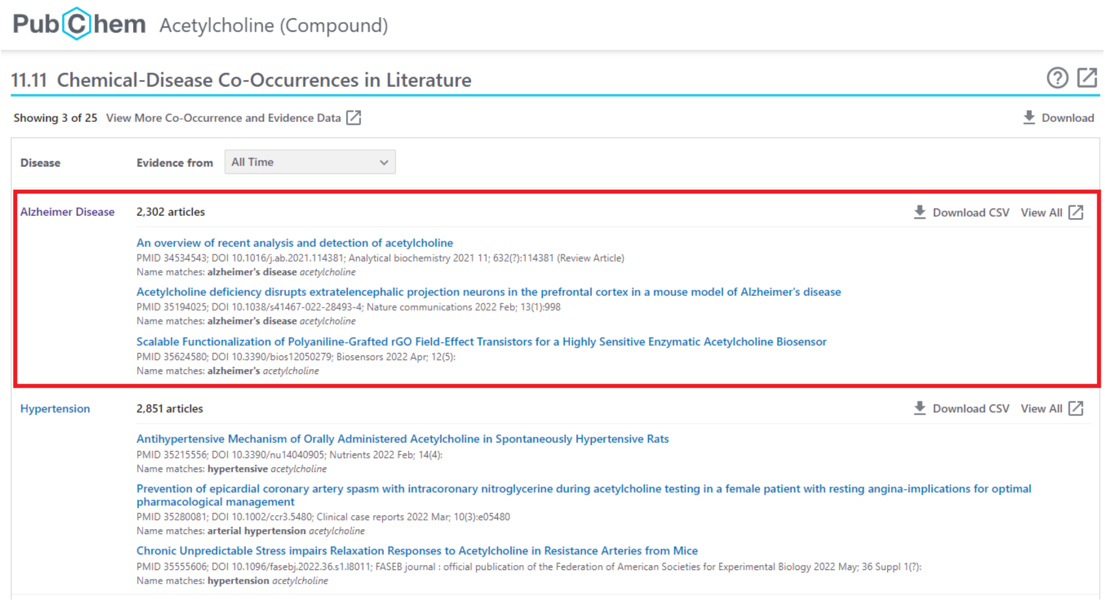

***Figure S4**: Screenshot of acetylcholine chemical- Disease Co-occurrences in Literature section in Pubchem. AD (outlined in red) is the first disease that appears in the section. Therefore, this chemical is included in "TOP1" list, as D00544 is the first neighbor. Source: https://pubchem.ncbi.nlm.nih.gov/compound/187#section=Chemical-Disease-Co-Occurrences-in-Literature (Accessed 13th March 2022).*

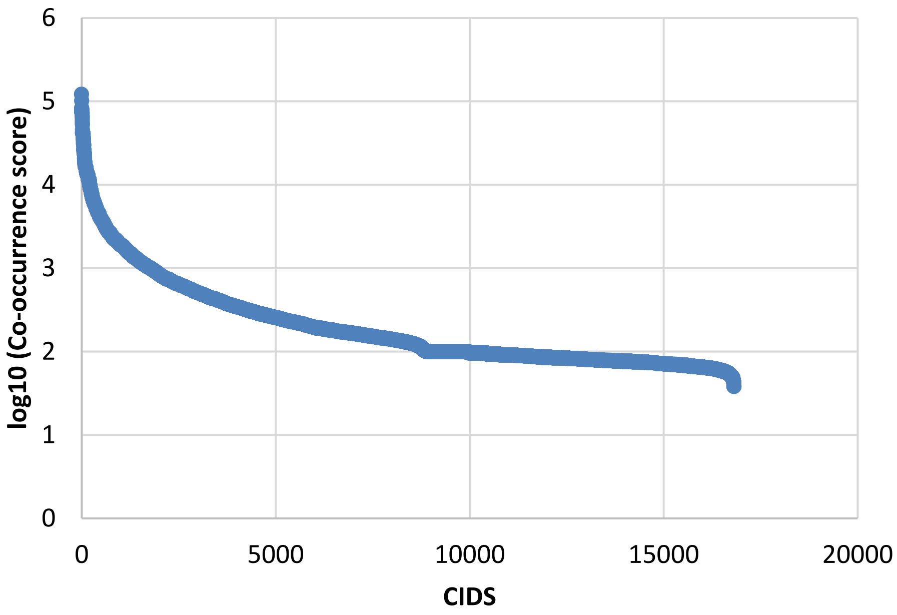

***Figure S5:** Scatter plot showing how the Co-occurrence Score is rapidly decreasing across all the CIDs present in the D00544 list.*

Section 1.5: Data processing

As described in the manuscript, both MS-DIAL[11] and patRoon[12,13] were employed to process the mzML files. MS-DIAL was used to perform non-target analysis via MSP-formatted libraries. MSMS-Public-Pos-VS17 and MSMS-Public-Neg-VS17 MSPs files were used for the (+) and (-) analysis, respectively. MS-DIAL parameters are summarized in the **Table S5**. Features without a tentative candidate via MS-DIAL were uploaded to MS-FINDER to annotate them via *in silico* structure fragmentation. MS-FINDER annotations were selected only in cases that improved the MS-DIAL annotations. **Figure S6** shows the workflow for the annotations with MS-DIAL and MS-FINDER schematically.

Additionally, patRoon was employed to perform both non-target and suspect screening. Features were extracted and grouped with *XCMS3*[14,15]. MS data was obtained using *mzR*[16]. Compound identification was performed by the *in silico* fragmenter MetFrag (MetFrag2.4.5-CL jar file). *PubChemLite*[17,18] (version 1.12.0) was used as a database, in addition to an AD-specific database and suspect lists described in the main text (*TOP1*, *SC20*, *AD-CTD* and *HMDB-CSF*). The same parameters were used to perform non-target and suspect screening. However, for the suspect screening positive and negative ionization modes were analyzed at the same time through the *makeSet* function of patRoon, while for the non-target screening the different modes were analyzed separately. After the analysis with MS-DIAL and patRoon peak intensities tables (csv formatted) were used to filter the features based on the QC samples (via *Tidyverse*[19] package). Features with relative standard deviation (RSD) > 30% and count ≤ 40% in the QC samples were discarded. Features with blank contribution >10% were removed. This step was performed separately for each of the QC types prepared in this study (QC-ND, QC-AD and QC-MCI), to avoid loss of features due to dilution. **Figure S3** shows the different QC samples employed for the data filtering schematically, as mentioned above.

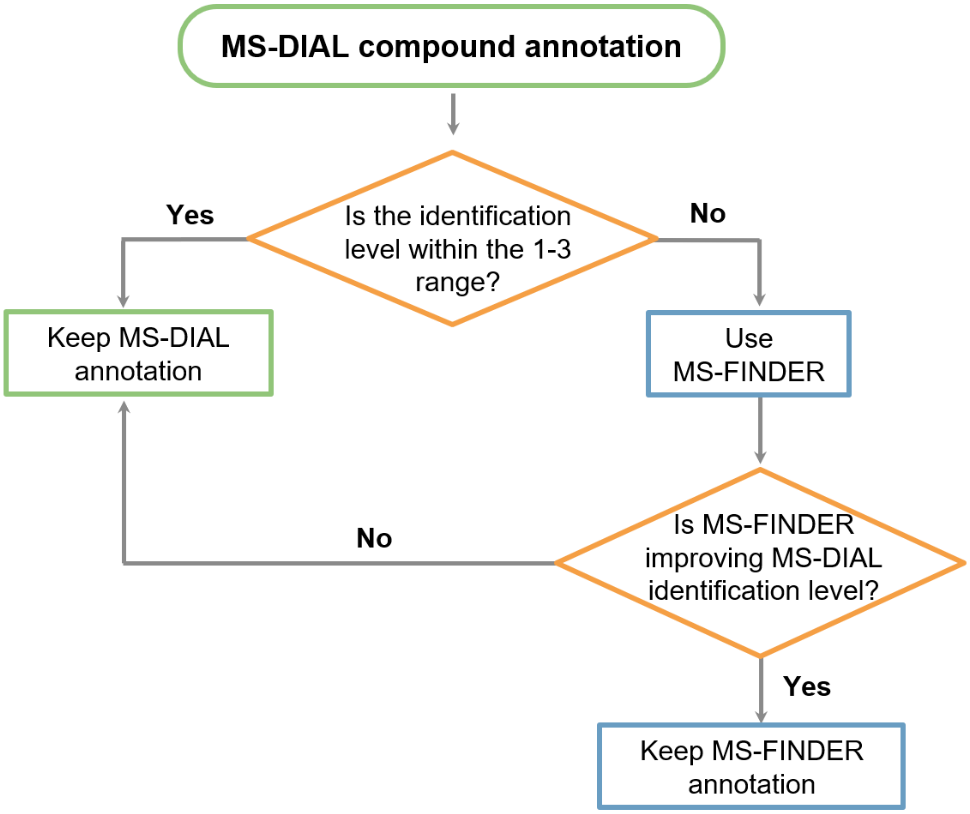

Figure S6: *Decision tree used for the annotation of the features identified with MS-DIAL.*

Peak intensity tables of the annotated features were pre-processed with MetaboAnalyst 5.0 by filtering (interquartile range option), normalization by sum, log transformation (base 10), and pareto scaling. Negative normalized peak intensities may arise from the combination of log transformation and pareto scaling. Pareto scaling uses the square root of the standard deviation as the scaling. These transformations (log and pareto scaling) are designed to enhance the data quality and facilitate downstream analysis[20]. Therefore, here a negative normalized peak intensity indicates that the intensity of the corresponding peak is lower than other samples in the dataset.

Level 1-3 compounds were classified using the HMDB disposition ontology[21,22], PubChem Classification Browser[23] Pathways information and/or literature associated with PubChem records via co-occurrence scores[24]. For the HMDB classification, the disposition ontology was explored, and compounds were categorized as either endogenous or exogenous. Some additional subcategories were included (*e.g.*, food, plant, microbial, toxin/pollutant, cosmetic, drug and

drug metabolite). For this step, filtered lists of metabolites were downloaded (https://hmdb.ca/metabolites) and merged into a single database (via R using the *Tidyverse*[19] package). The next figure shows an overview of the steps followed for the database curation and mapping with the annotated compounds. Details about the database curation and alluvial plots are shared in the GitLab repository[6].

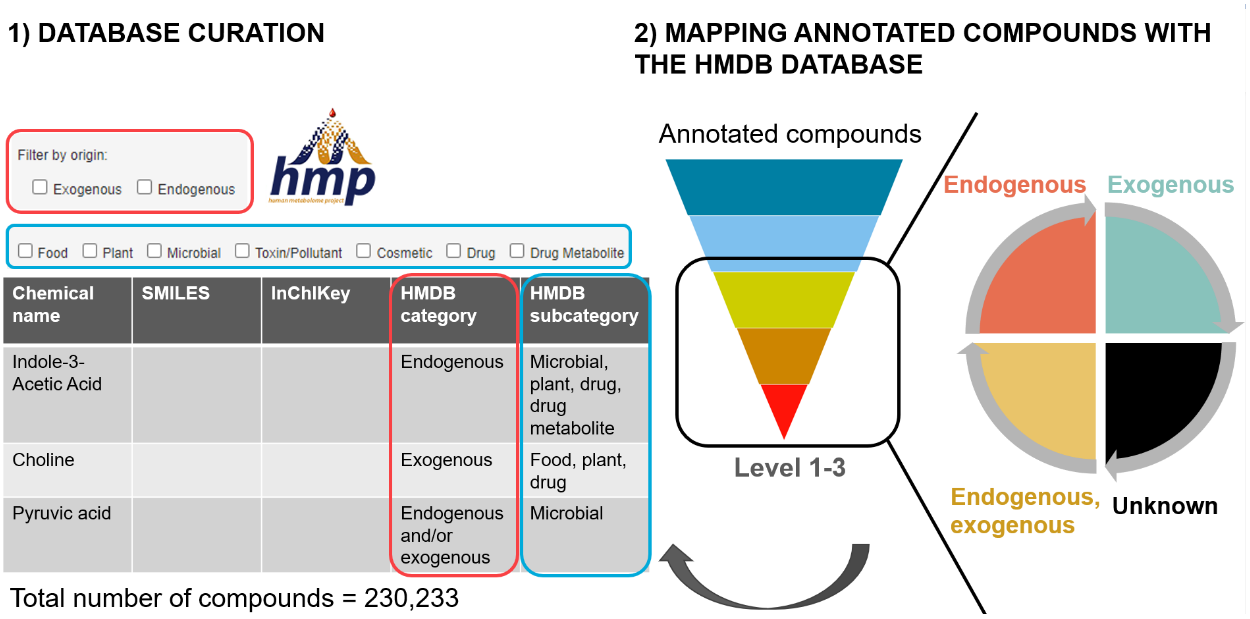

Figure S7: *Overview of the steps followed for the HMDB curation (1) as well as mapping with the annotated compounds via MS-DIAL and patRoon (2). Information about the HMDB compounds was obtained from:* https://hmdb.ca/metabolites.

Section 2: Results and discussion

Section 2.1: Non-target characterization of CSF in MCI and AD

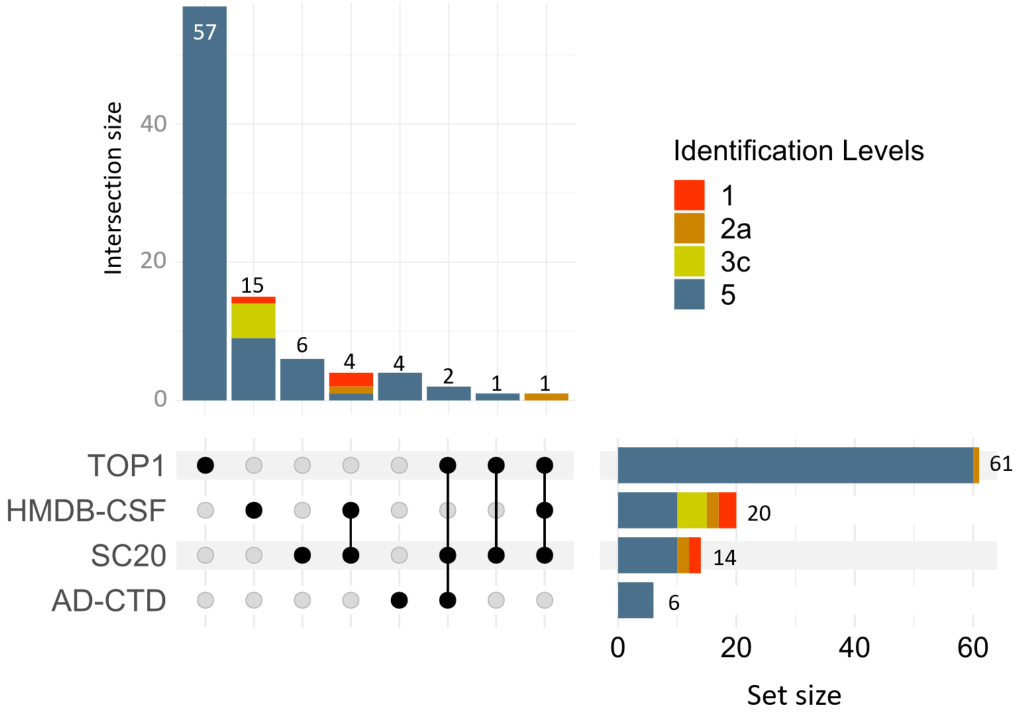

Figure S8: *UpSet plot representing the number of annotated features in each suspect lists plus overlap across lists by RPLC. The fill of the bars represents the identification level of each of the features. Features identified by (+) and (-) were combined, for simplicity (duplicates were removed).*

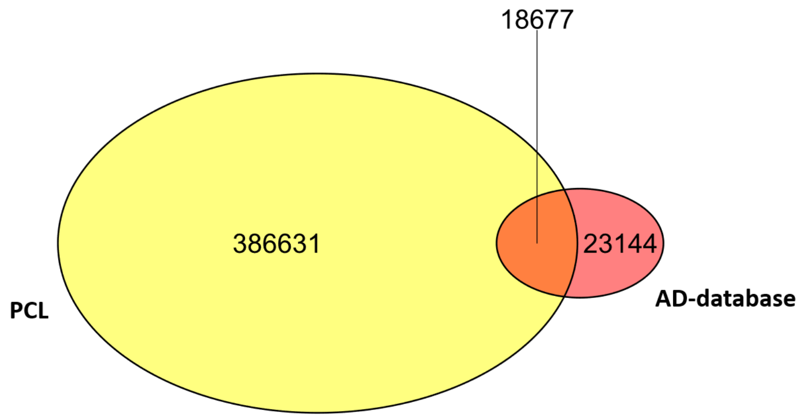

Figure S9: *Venn plot showing the overlap between the AD-database and PCL.*

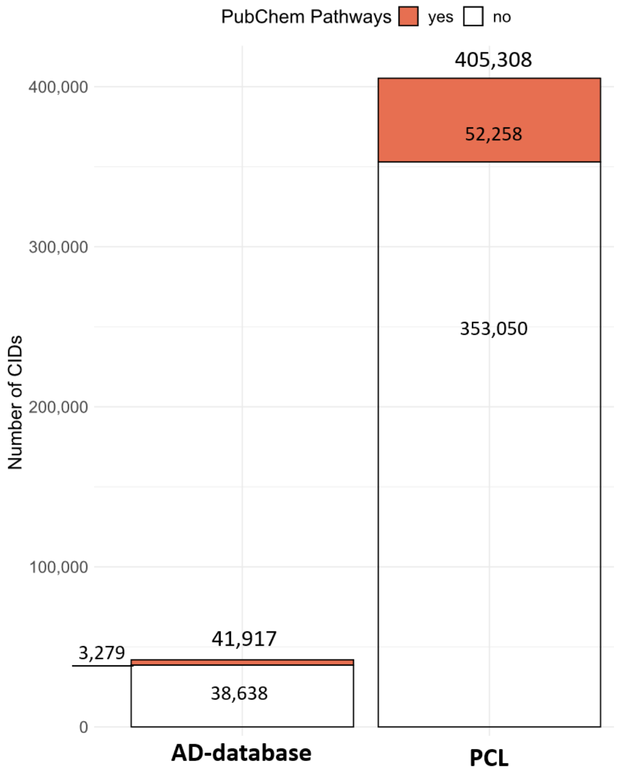

Figure S10: Bar plot showing the total number of CIDs in the AD-database and PCL as well as the CIDs that contains Pathways information, suggesting an endogenous source.

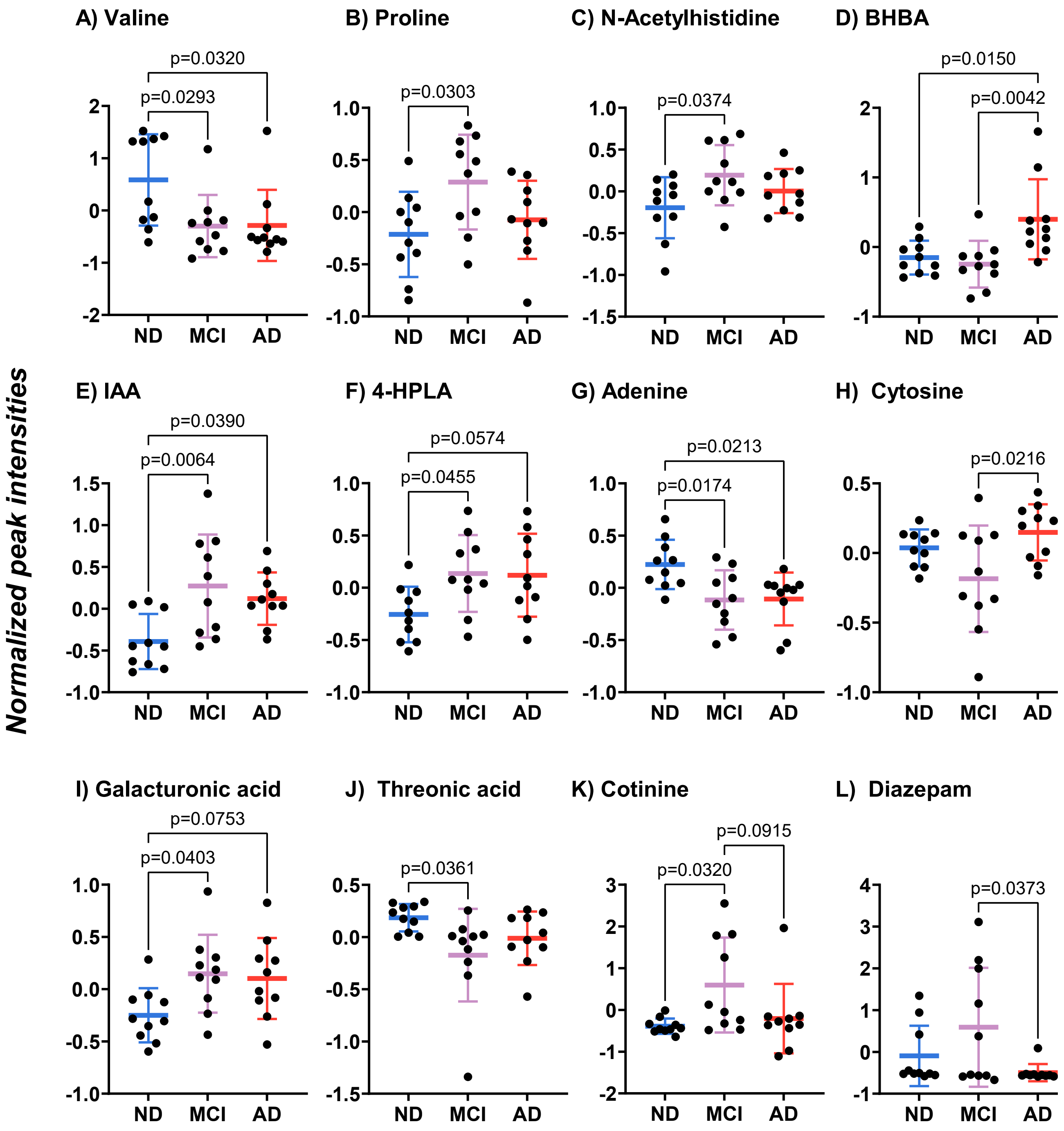

Figure S11: *Scatter dot plots showing mean ± standard deviation (SD) of the normalized peak intensities across groups of (A) valine, (B) proline, (C) N-acetylhistidine, (D) 3-hydroxybutanoic acid (BHBA), (E) Indole-3-acetic acid (IAA), (F) 4-Hydroxyphenyllactic acid (4-HPLA), (G) adenine, (H) cytosine, (I) galacturonic acid, (J) threonic acid, (K) cotinine and (L) diazepam. p= Tukey's HSD post-hoc p-value. Note that p-values <0.1 are displayed although only p-values <0.05 are considered statistically significant in this work.*

Section 2.2: Target study of BAs in CSF of MCI and AD

Cholesterol metabolism is thought to play a role in AD pathology[25–28]. The brain uses the alternative and neural pathway of BAs synthesis, and not the classical pathway, to clear cholesterol[29,30]. The intermediates of the alternative pathway -3β-OH-5-cholestenoic acid (3β-OH-5-COA), 3β-7α-DiOH-5-cholestenoic acid (3β-7α-DiOH-5-COA), and 7α-hydroxy-3-oxo-4-cholestenoic acid (7-HOCA)- exhibited higher but non-significant concentrations in the MCI and AD groups compared with the ND (**Figure S12**). This observation may be attributed to the dysregulation of cholesterol homeostasis in AD, potentially resulting in an excess of cholesterol in the brain[28].

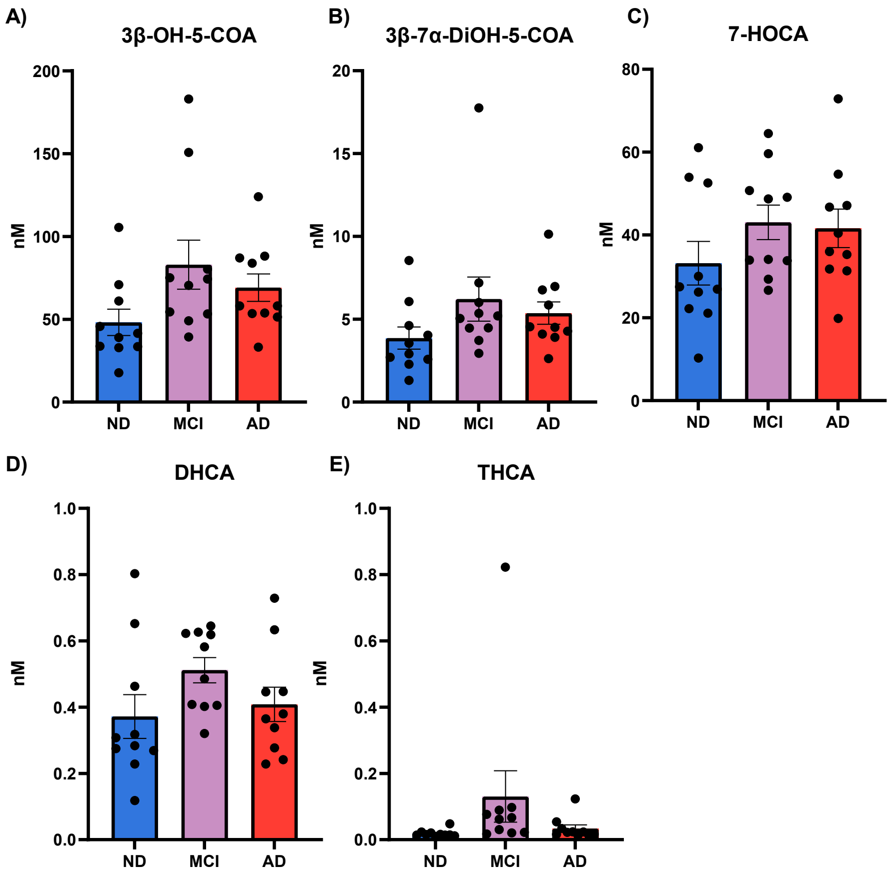

Figure S12: *Bar plots showing the mean concentration with SEM of* ***BA precursors****. (A) 3β-OH-5-cholestenoic acid (3β-OH-5-COA), (B) 3β-7α-DiOH-5 cholestenoic acid (3β-7α-DiOH-5-COA), (C) 7α-Hydroxy-3-oxo-4-cholestenoic acid (7-HOCA), (D) 3α,7α-dihydroxycholestanoic acid (DHCA), (E) 3α,7α,12α-trihydroxycholestanoic acid (THCA). Plots display bile acid concentrations among groups, encompassing all 30 samples (samples below LOD were not excluded). SEM: Standard Error of the Mean.*

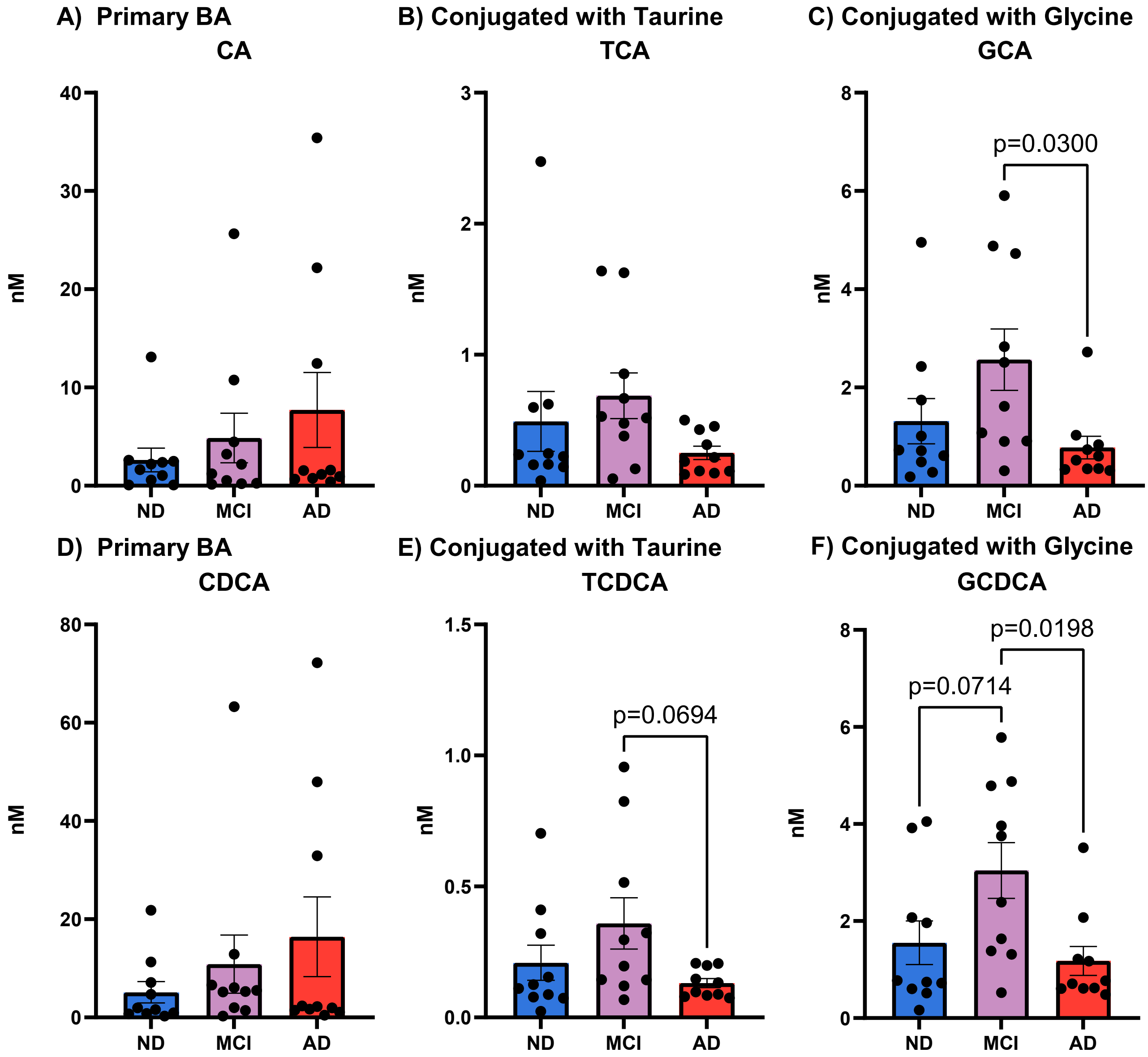

Figure S13: *Bar plots showing the mean concentration with SEM of the* ***primary conjugated and unconjugated BAs****. (A) Cholic acid (CA), (B) Taurocholic acid (TCA), (C) Glycocholic acid (GCA), (D) Chenodeoxycholic acid (CDCA), (E) Taurochenodeoxycholic acid (TCDCA), (F) Glycochenodeoxycholic acid (GCDCA). p= Tukey's HSD post-hoc p-value. Note that p-values <0.1 are displayed although only p-values <0.05 are considered statistically significant in this work. Plots display bile acid concentrations among groups, encompassing all 30 samples (samples below LOD were not excluded). SEM: Standard Error of the Mean.*

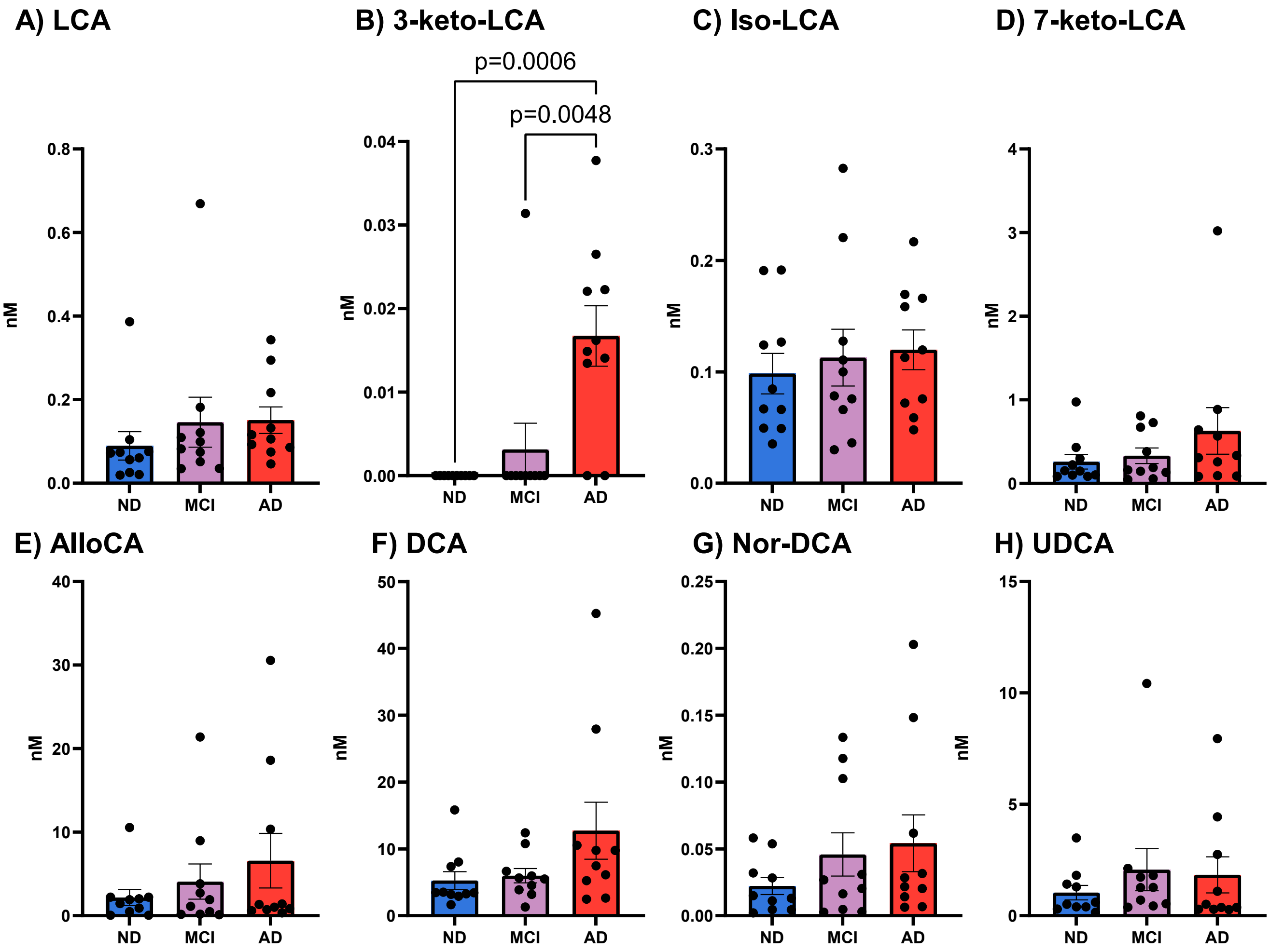

Figure S14: *Bar plots showing the mean concentration with SEM of the* ***secondary unconjugated BAs****. (A) Lithocholic acid (LCA), (B) Dehydrolithocholic acid (3-keto-LCA) (C) Isolithocholic acid (Iso-LCA), (D) 7-Ketolithocholic acid (7-keto-LCA), (E) Allocholic acid (AlloCA), (F) Deoxycholic acid (DCA), (G) Nordeoxycholic acid (Nor-DCA) and (H) Ursodeoxycholic acid (UDCA). Plots display bile acid concentrations among groups, encompassing all 30 samples (samples below LOD were not excluded). p= Tukey's HSD post-hoc p-value. SEM: Standard Error of the Mean.*

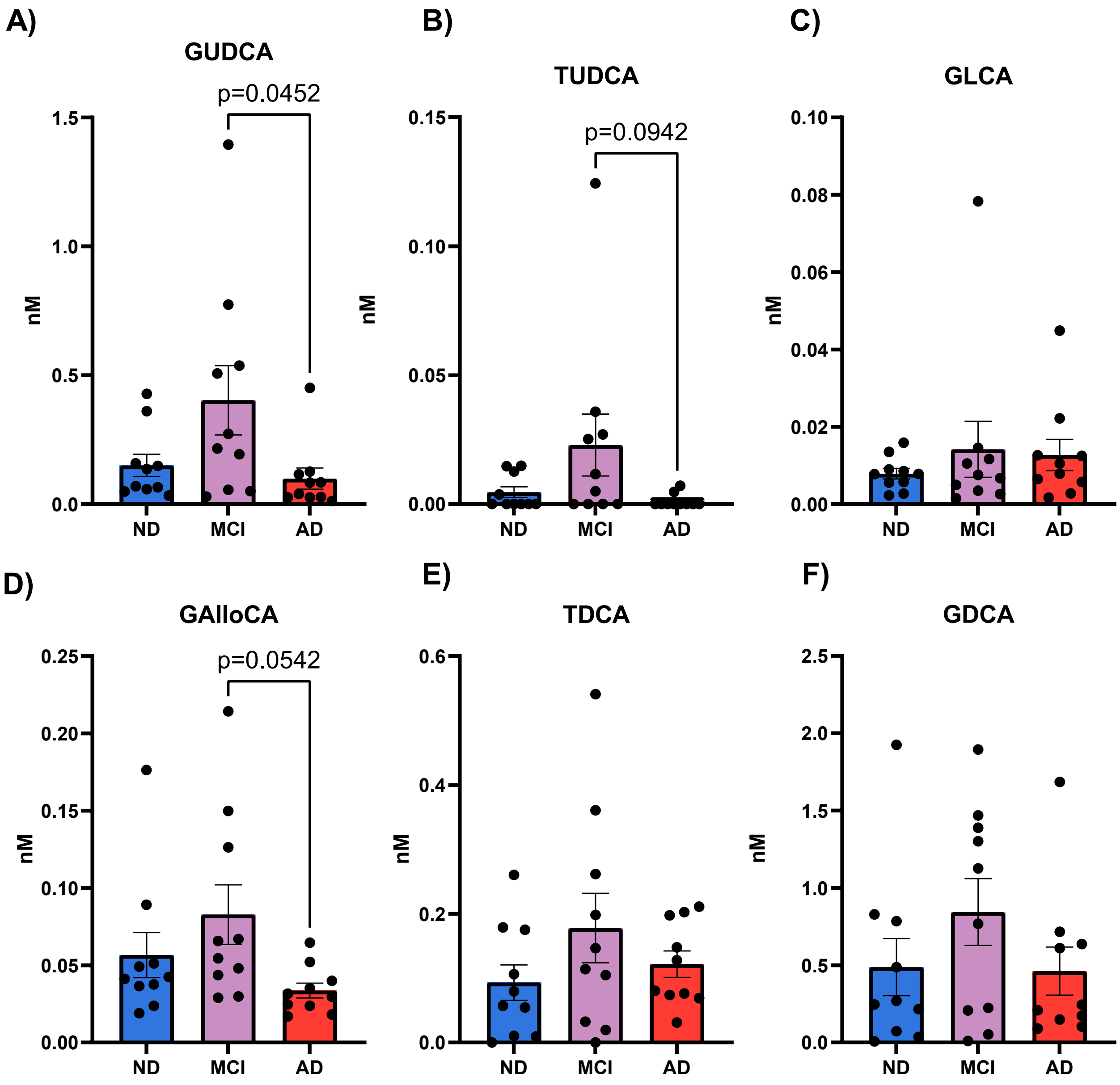

Figure S15: *Bar plots showing the mean concentration with SEM of the* ***secondary conjugated BAs*** *(A) Glucoursodeoxycholic acid (GUDCA), (B) Tauroursodeoxycholic acid (TUDCA) (C) Glycolithocholic acid (GLCA), (D) Glycoallocholic acid (GAlloCA), (E) Taurodeoxycholic acid (TDCA), (F) Glycodeoxycholic acid (GDCA). p= Tukey's HSD post-hoc p-value. Note that p-values <0.1 are displayed although only p-values <0.05 are considered statistically significant in this work. Plots display bile acid concentrations among groups, encompassing all 30 samples (samples below LOD were not excluded). SEM: Standard Error of the Mean.*

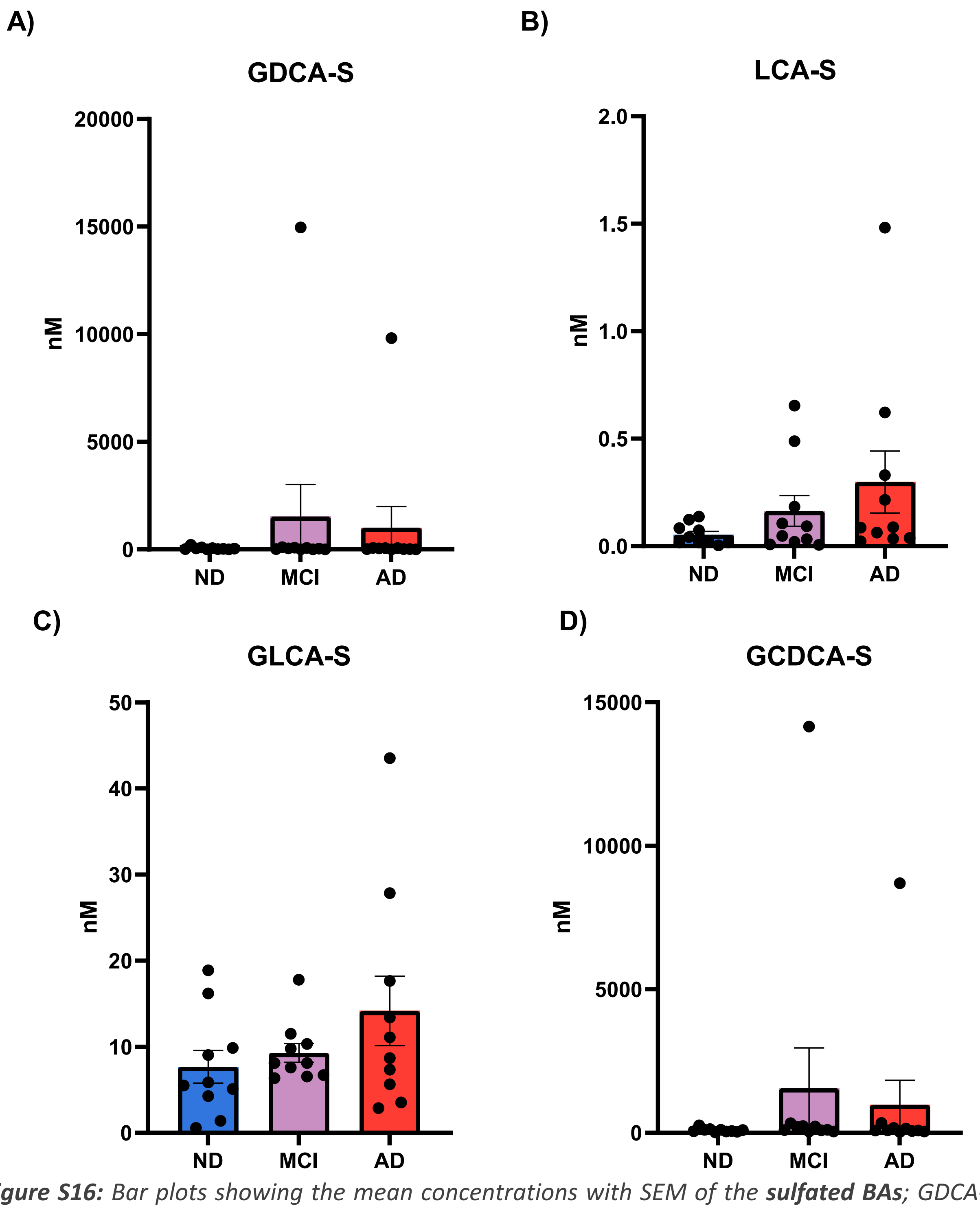

Figure S16: *Bar plots showing the mean concentrations with SEM of the* ***sulfated BAs****; GDCA-S (A), LCA-S (B), GLCA-S (C) and GCDCA-S (D). SEM: Standard Error of the Mean. Plots display bile acid concentrations among groups, encompassing all 30 samples (samples below LOD were not excluded).*

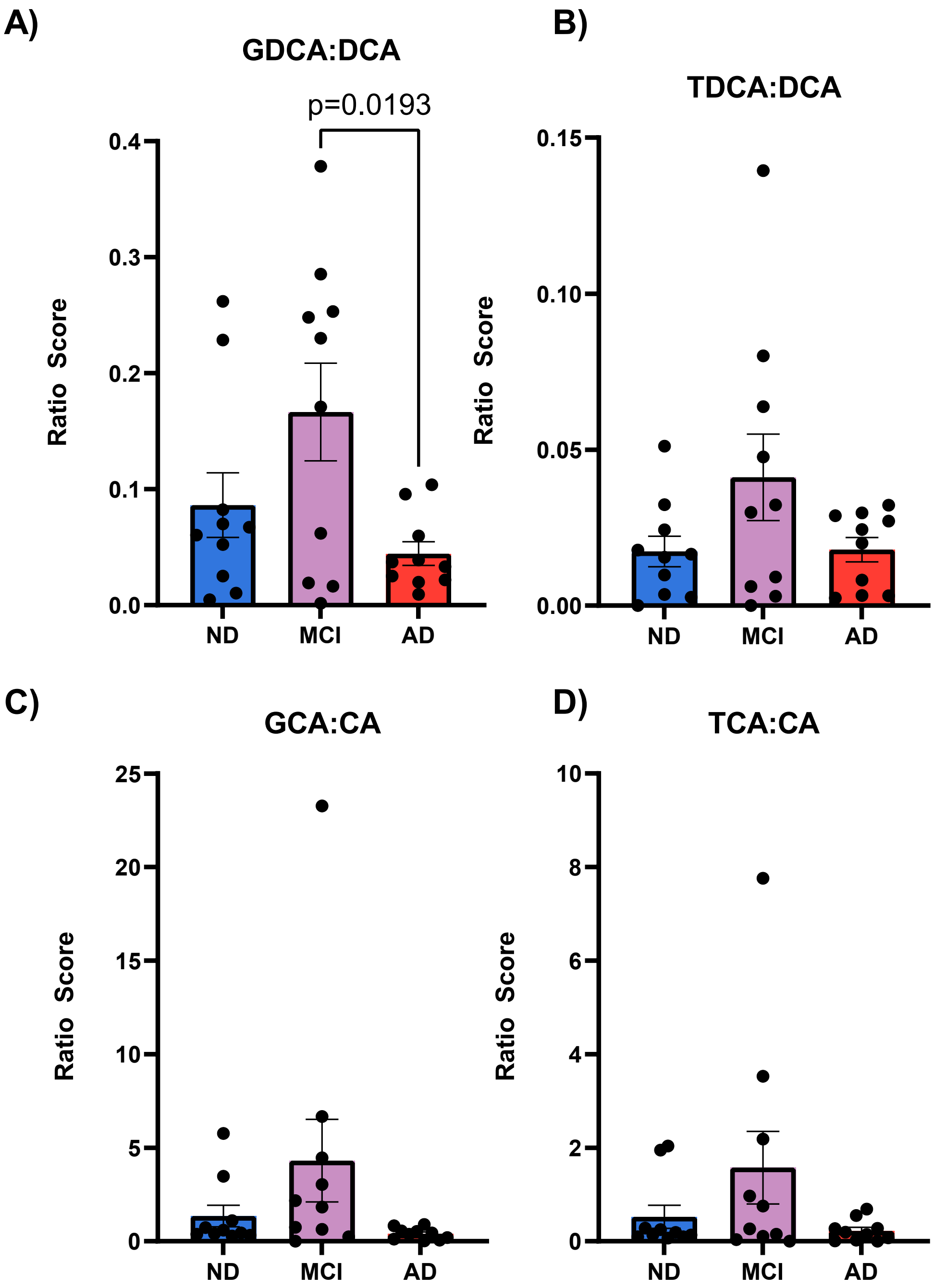

Figure S17: *Bar plots showing the mean ratio score with SEM of GDCA:DCA (A), TDCA:DCA (B), GCA:CA (C), and TCA:CA (D). p= Tukey's HSD post-hoc p-value. SEM: Standard Error of the Mean.*

Section 3: Future Perspectives

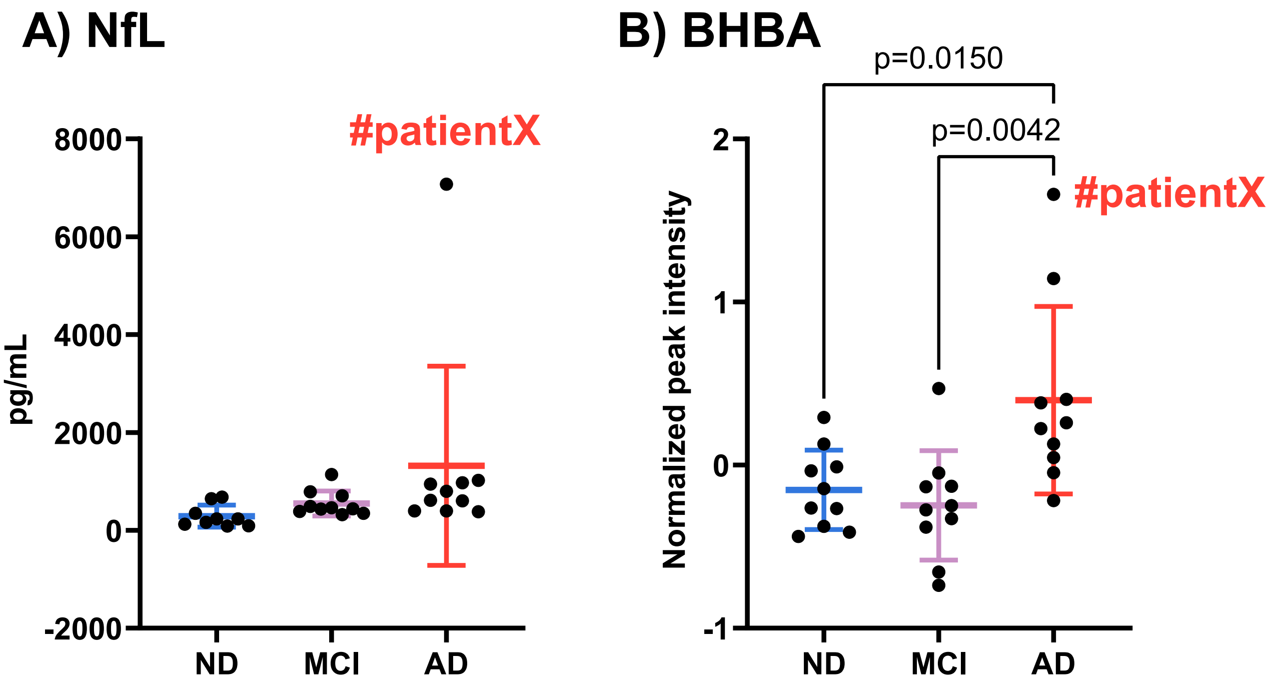

Figure S18: *Scatter plots showing mean ± SD of the concentration of NfL (A) and normalized peak intensities of BHBA (B). Note that the outlier in the AD group is the same in both cases (#Xpatient). p= Tukey's HSD post-hoc p-value.*

References

1. Song Z, Wang M, Zhu Z, Tang G, Liu Y, Chai Y. Optimization of pretreatment methods for cerebrospinal fluid metabolomics based on ultrahigh performance liquid chromatography/mass spectrometry. *Journal of Pharmaceutical and Biomedical Analysis*. 2021;197:113938. doi:10.1016/j.jpba.2021.113938

2. Broadhurst D, Goodacre R, Reinke SN, et al. Guidelines and considerations for the use of system suitability and quality control samples in mass spectrometry assays applied in untargeted clinical metabolomic studies. *Metabolomics*. 2018;14(6):72. doi:10.1007/s11306-018-1367-3

3. Frigerio G, Moruzzi C, Mercadante R, Schymanski EL, Fustinoni S. Development and Application of an LC-MS/MS Untargeted Exposomics Method with a Separated Pooled Quality Control Strategy. *Molecules*. 2022;27(8):2580. doi:10.3390/molecules27082580

4. PubChem Identifier Exchange Service. Accessed September 8, 2022. https://pubchem.ncbi.nlm.nih.gov/idexchange/idexchange.cgi

5. Szöcs E, Allaway R, Muench D, et al. webchem: Chemical Information from the Web. Published online June 15, 2022. Accessed September 28, 2022. https://CRAN.R-project.org/package=webchem

6. Environmental Cheminformatics / AD-CSF · GitLab. GitLab. Published May 1, 2023. Accessed June 12, 2023. https://gitlab.lcsb.uni.lu/eci/AD-CSF

7. Zaslavsky L, Cheng T, Gindulyte A, et al. Discovering and Summarizing Relationships Between Chemicals, Genes, Proteins, and Diseases in PubChem. *Front Res Metr Anal*. 2021;6:689059. doi:10.3389/frma.2021.689059

8. Wishart DS, Lewis MJ, Morrissey JA, et al. The human cerebrospinal fluid metabolome. *J Chromatogr B Analyt Technol Biomed Life Sci*. 2008;871(2):164-173. doi:10.1016/j.jchromb.2008.05.001

9. CSF Metabolome. Accessed November 4, 2022. https://csfmetabolome.ca/

10. Andújar BT, Mary A, Cheng T, et al. PubChem lists to Study the Exposome of Mild Cognitive Impairment and Alzheimer's Disease on Cerebrospinal Fluid. Published online June 7, 2023. doi:10.5281/zenodo.8014420

11. Tsugawa H, Cajka T, Kind T, et al. MS-DIAL: data-independent MS/MS deconvolution for comprehensive metabolome analysis. *Nat Methods*. 2015;12(6):523-526. doi:10.1038/nmeth.3393

12. Helmus R, ter Laak TL, van Wezel AP, de Voogt P, Schymanski EL. patRoon: open source software platform for environmental mass spectrometry based non-target screening. Published online January 2021. doi:10.1186/s13321-020-00477-w

13. Helmus R, Velde B van de, Brunner AM, Laak TL ter, Wezel AP van, Schymanski EL. patRoon 2.0: Improved non-target analysis workflows including automated transformation product screening. *Journal of Open Source Software*. 2022;7(71):4029. doi:10.21105/joss.04029

14. Neumann S. The xcms package (version >= 3). Published online February 28, 2022. Accessed March 11, 2022. https://github.com/sneumann/xcms

15. Smith CA, Tautenhahn R, Neumann S, et al. xcms: LC-MS and GC-MS Data Analysis. Published online 2022. doi:10.18129/B9.bioc.xcms

16. Fischer B, Neumann S, Gatto L, Kou Q, Rainer J. mzR: parser for netCDF, mzXML, mzData and mzML and mzIdentML files (mass spectrometry data). Published online 2022. doi:10.18129/B9.bioc.mzR

17. Schymanski EL, Kondić T, Neumann S, Thiessen PA, Zhang J, Bolton EE. Empowering large chemical knowledge bases for exposomics: PubChemLite meets MetFrag. *J Cheminform*. 2021;13(1):19. doi:10.1186/s13321-021-00489-0

18. Bolton E, Schymanski E, Kondic T, Thiessen P, Zhang J. PubChemLite for Exposomics. Published online October 31, 2020. doi:10.5281/zenodo.4183801

19. Wickham H, Averick M, Bryan J, et al. Welcome to the Tidyverse. *Journal of Open Source Software*. 2019;4(43):1686. doi:10.21105/joss.01686

20. van den Berg RA, Hoefsloot HC, Westerhuis JA, Smilde AK, van der Werf MJ. Centering, scaling, and transformations: improving the biological information content of metabolomics data. *BMC Genomics*. 2006;7:142. doi:10.1186/1471-2164-7-142

21. Wishart DS, Feunang YD, Marcu A, et al. HMDB 4.0: the human metabolome database for 2018. *Nucleic Acids Res*. 2018;46(Database issue):D608-D617. doi:10.1093/nar/gkx1089

22. Human Metabolome Database. Accessed December 5, 2023. https://hmdb.ca/

23. Kim S, Chen J, Cheng T, et al. PubChem 2023 update. *Nucleic Acids Research*. 2023;51(D1):D1373-D1380. doi:10.1093/nar/gkac956

24. Zaslavsky L, Cheng T, Gindulyte A, et al. Discovering and Summarizing Relationships Between Chemicals, Genes, Proteins, and Diseases in PubChem. *Front Res Metr Anal*. 2021;6:689059. doi:10.3389/frma.2021.689059

25. Griffiths WJ, Abdel-Khalik J, Yutuc E, et al. Concentrations of bile acid precursors in cerebrospinal fluid of Alzheimer's disease patients. *Free Radic Biol Med*. 2019;134:42-52. doi:10.1016/j.freeradbiomed.2018.12.020

26. MahmoudianDehkordi S, Arnold M, Nho K, et al. Altered bile acid profile associates with cognitive impairment in Alzheimer's disease—An emerging role for gut microbiome. *Alzheimer's & Dementia*. 2019;15(1):76-92. doi:10.1016/j.jalz.2018.07.217

27. Shobab LA, Hsiung GYR, Feldman HH. Cholesterol in Alzheimer's disease. *The Lancet Neurology*. 2005;4(12):841-852. doi:10.1016/S1474-4422(05)70248-9

28. Hughes TM, Rosano C, Evans RW, Kuller LH. Brain Cholesterol Metabolism, Oxysterols, and Dementia. *J Alzheimers Dis*. 2013;33(4):891-911. doi:10.3233/JAD-2012-121585

29. Baloni P, Funk CC, Yan J, et al. Metabolic Network Analysis Reveals Altered Bile Acid Synthesis and Metabolism in Alzheimer's Disease. *Cell Reports Medicine*. 2020;1(8):100138. doi:10.1016/j.xcrm.2020.100138

30. Monteiro-Cardoso VF, Corlianò M, Singaraja RR. Bile Acids: A Communication Channel in the Gut-Brain Axis. *Neuromol Med*. 2021;23(1):99-117. doi:10.1007/s12017-020-08625-z
